# The palliative care needs of people with multiple long-term conditions and their family caregivers: a scoping review of mixed study designs

**DOI:** 10.1093/ageing/afag324

**Published:** 2026-11-13

**Authors:** Felicity Dewhurst, Mark Adley, Owen Finney, Alex Inskip, Eugenie Evelynne Johnson, Sarah Bowers, Lucy Robinson, Kathryn Elliott-Smith, Charlotte Stenson, Tafadzwa Patience Kunonga, Barbara Hanratty

**Affiliations:** Newcastle University—Population Health Sciences Institute, Newcastle Upon Tyne, United Kingdom of Great Britain and Northern Ireland; Newcastle University—Population Health Sciences Institute, Newcastle Upon Tyne, United Kingdom of Great Britain and Northern Ireland; North East Ambulance Service Nhs Foundation Trust, Newcastle Upon Tyne, United Kingdom of Great Britain and Northern Ireland; Newcastle University—National Institute for Health and Care Research (NIHR) Innovation Observatory, Newcastle Upon Tyne, United Kingdom of Great Britain and Northern Ireland; Newcastle University—Population Health Sciences Institute, Newcastle Upon Tyne, United Kingdom of Great Britain and Northern Ireland; Newcastle University—National Institute for Health and Care Research (NIHR) Innovation Observatory, Newcastle Upon Tyne, United Kingdom of Great Britain and Northern Ireland; University of St Andrews—School of Medicine, St Andrews, United Kingdom of Great Britain and Northern Ireland; Newcastle University—School of Psychology, Newcastle Upon Tyne, United Kingdom of Great Britain and Northern Ireland; Newcastle University—Population Health Sciences Institute, Newcastle Upon Tyne, United Kingdom of Great Britain and Northern Ireland; Newcastle University—Population Health Sciences Institute, Newcastle Upon Tyne, United Kingdom of Great Britain and Northern Ireland; Newcastle University—Population Health Sciences Institute, Newcastle Upon Tyne, United Kingdom of Great Britain and Northern Ireland; Newcastle University—Population Health Sciences Institute, Newcastle Upon Tyne, United Kingdom of Great Britain and Northern Ireland

**Keywords:** multiple long-term conditions, multimorbidity, palliative care, end-of-life care, equitable care, older people

## Abstract

**Background:**

As populations age, most deaths occur in people with multiple long-term conditions (MLTC). When decline is driven by coexisting conditions, the palliative phase and associated needs often go unrecognised. Improving palliative care for this group is an international priority, yet no recent review has described their needs at the end of life.

**Objective:**

To map the extent, range and nature of evidence on the palliative care needs of people with MLTC and their family caregivers, describe its characteristics and identify knowledge gaps.

**Design:**

Scoping review of quantitative, qualitative and mixed-methods primary studies.

**Eligibility criteria:**

Adults aged ≥50 years with ≥2 long-term conditions plus indicators of advanced disease or poor prognosis. Single-condition studies, early-stage MLTC, non-disaggregated data, disease-modifying interventions and non-primary research were excluded.

**Sources of Evidence:**

MEDLINE, EMBASE, CINAHL, Scopus and PsycINFO, searched from inception to 8 September 2025.

**Charting Methods:**

Records were dual-screened and charted by design, setting and established conceptual frameworks of domains of need.

**Results:**

Of 7568 records, 90 studies were included. Four domains were identified: (i) comprehensive support needs in the context of functional decline; (ii) proactive care needs shaped by limited death literacy and prognostic uncertainty; (iii) systems-level needs for coordination and collaboration; and (iv) compounded needs arising from inequality (diagnostic/sociodemographic).

**Conclusion:**

Older adults with MLTC face complex, often unmet needs, requiring support for functional decline, equitable proactive care, coordinated systems and improved death literacy. Palliative care must be integrated, responsive to MLTC realities and guided by what matters most to patients and caregivers.

## Key points

- Older adults with multiple long-term conditions (MLTC) live with interdependent, and often unaddressed, palliative needs
- Functional decline was the most frequently reported pathway through which care needs manifest and care priorities are set by people with MLTC
- Triggered, evolving advance care planning delivered through multi-agency working is needed to enable goal-concordant care
- Equitable access to palliative care should be explicitly considered, and delivered though culturally competent practice

## Introduction

As populations age, most deaths occur among older adults with multiple long-term conditions (MLTC) [1–3]. Advanced MLTC is characterised by interacting disease processes, functional decline and high symptom burden; with prolonged, episodic trajectories in which acute exacerbations are followed by incomplete recovery, that complicate recognition of the palliative phase and timely care planning [4–6]. Palliative care aims to improve quality of life for people with life-limiting illness and their family caregivers [7, 8]. However, models largely developed for cancer may be poorly suited to the less predictable trajectories and prolonged needs associated with advanced MLTC [9–11]. Despite recognised palliative care needs, people with MLTC, without a predominant condition, are less likely to access services. This is particularly true for people from socioeconomically disadvantaged or minoritised groups, which contributes to persistent inequalities in ageing and end-of-life outcomes [12–17].

Prior reviews have examined palliative care needs in adjacent populations, including people with frailty [18, 19] and single-condition or disease-specific groups, but these have not comprehensively mapped need across multiple domains for people with MLTC as a distinct clinical population. Reviews of integrated care models for multimorbidity and older people [20, 21] have similarly not addressed the palliative phase. This scoping review therefore aims to address that gap by mapping the extent, range and nature of evidence on the palliative care needs of people with MLTC and their family caregivers, describing its characteristics, and identifying knowledge gaps.

## Methods

### Full details of the review methods are provided in Appendix 8 of the supplementary data section

#### Design and reporting

A scoping review was selected because the review question concerned the extent, range and nature of a large and heterogeneous body of evidence, and the aim was to identify gaps and inform future practice, policy-relevant research and more focused synthesis [22]. The review adhered to Preferred Reporting Items for Systematic Reviews and Meta-Analyses—Scoping Review extension (PRISMA-ScR) guidelines [23, 24] (Appendix 1). The protocol was registered with PROSPERO (registration number: CRD42024510220), with deviations from the protocol and reasons for deviations reported in Appendix 2. Quantitative, qualitative and mixed-methods primary studies were eligible. Critical appraisal was not undertaken, consistent with scoping review guidance; the review therefore describes what has been reported and does not appraise the strength or reliability of that reporting [25].

#### Eligibility criteria

Studies were eligible if they reported original empirical research (quantitative, qualitative or mixed methods) on the needs of adults aged 50 years and over with two or more long-term conditions and at least one indicator of a palliative phase, or on the needs of their adult caregivers, in any setting or country [5, 10, 11, 26–29]. The age threshold was set below the conventional 65 years so as not to exclude populations in whom MLTC develops earlier: onset occurs 10 to 15 years earlier in the most deprived compared with the most affluent areas [30], and the median age of onset has fallen while socioeconomic inequalities have widened, particularly below 65 years [31]. Study eligibility criteria, including indicators of the palliative phase, are detailed in Appendix 3.

#### Search strategy

MEDLINE, EMBASE, CINAHL, Scopus and PsycINFO were searched from inception to 8 September 2025. Search strategies were developed with an information specialist. Search strings for each database, with the date run and records retrieved, are provided in Appendix 4.

#### Study selection

Records were de-duplicated in Endnote [32] and imported into Rayyan [33], where all underwent dual independent screening at title/abstract and full-text stages. Full texts were retrieved via institutional subscriptions and library document supply, with interlibrary loan and direct requests to authors where necessary; five records could not be obtained (Fig. 1). Discrepancies were resolved through discussion and adjudication by a third reviewer (B.H.) [23].

**Figure 1:**
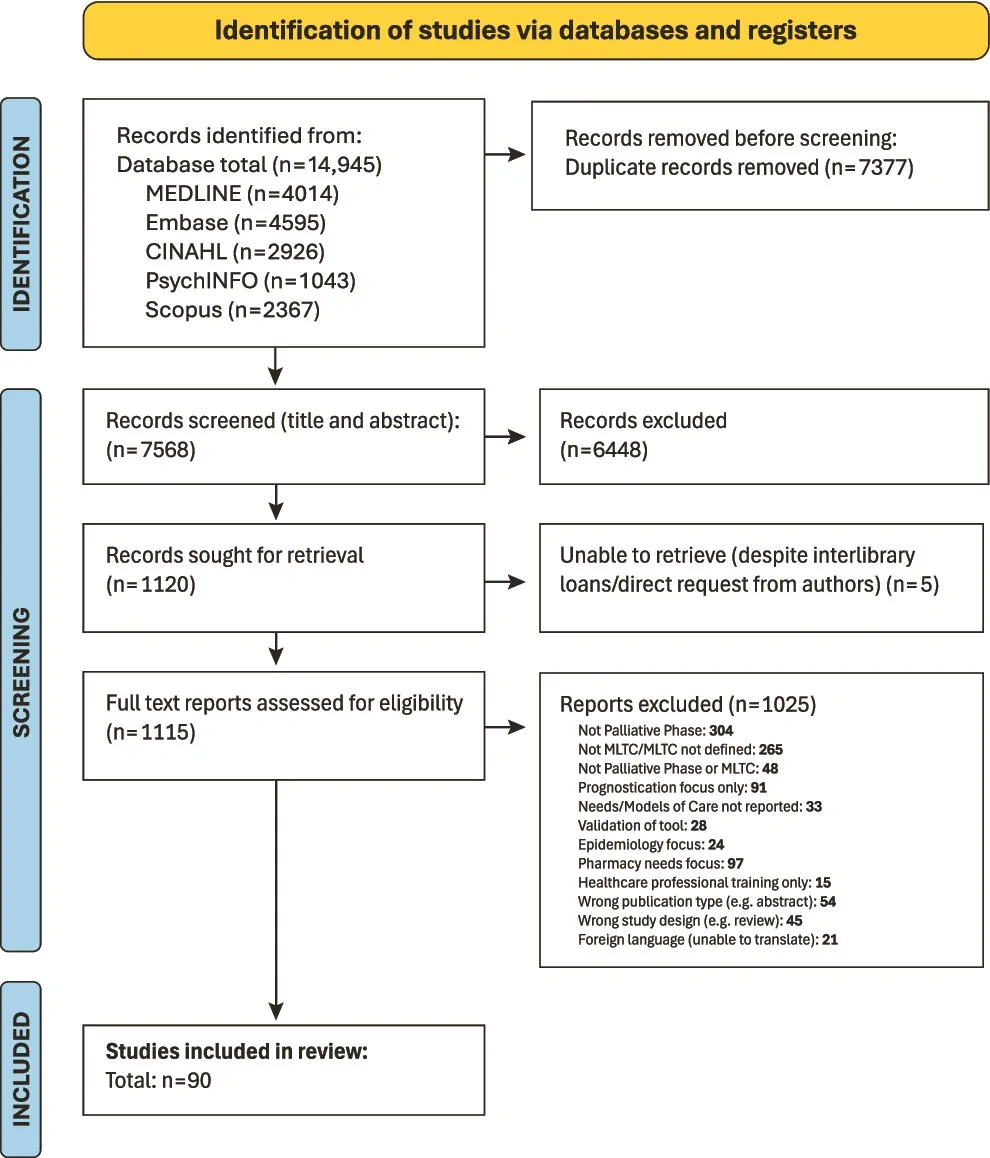
Study Selection Flowchart.

#### Data charting

Data were extracted and charted by O.F. and M.A., and verified by F.D. A standardised data extraction form developed specifically for this review was used. The form was piloted on a sample of included studies by two reviewers, with modifications made to improve clarity and completeness before full data extraction commenced. Charting captured study characteristics, population, setting, the MLTC definition used and the presence or absence of each of the 15 needs subdomains.

#### Data synthesis

Findings from each included study, whether reported as themes, survey responses, instrument scores or routinely collected data, were coded inductively to descriptive statements of need, compared across studies and grouped into 15 subdomains; these were refined iteratively and then applied to all 90 studies to produce the coverage matrix in Appendix 6. All 90 studies contributed to the synthesis and to the coverage matrix, and none was excluded; those named in the text were selected to illustrate diverse designs, settings and illness trajectories.

Quantitative and qualitative findings were integrated using a convergent integrated approach, in which quantitative results were narratively described and combined with qualitative findings against a common set of subdomains [34, 35]. The 15 subdomains were then grouped into four overarching domains, using published conceptual frameworks in geriatrics and palliative care as an organising lens, with a worked example of the analytical synthesis process in Appendix 7.

## Results

### Study selection

Searches yielded 7568 unique records; 6448 were excluded at title/abstract stage, leaving 1120 records sought for retrieval, of which 1115 full texts were assessed. Ninety studies met the eligibility criteria and were included (Fig. 1).

### Characteristics of included studies

The 90 studies included in this scoping review (Table 1) were conducted in more than 24 countries spanning 2005–25. Key data are charted in Table 2. The marked increase in published articles post-2019 suggests a growing recognition of this field of study, with 2025 already the single largest year (*n* = 17).

**Table 1:** Summary of key characteristics of included studies (n/a = not reported, or not able to extrapolate MLTC population data).

Author(s)	Country	Design (grouped)^a^	Setting (grouped)^a^	Total MLTC patients	Female	Mean age	Race/ethnicity collected/analysed^b^	Comorbidity measure used	Study aim (grouped)^a^
Abedini *et al.* [36]	USA^c^	Retrospective cohort/record-based	Mixed settings	2896	1447	77.9^d^	Collected only	Multimorbidity-Weighted Index	Describe patient needs, symptoms, burden/experience
Almagro *et al.* [37]	Spain	Prospective cohort/longitudinal	Inpatient hospital	843	428	82.4	No collection/analysis	Charlson Comorbidity Index	Service utilisation or healthcare outcomes
Alnajar *et al.* [38]	Jordan	Cross-sectional	Inpatient hospital	458	187	n/a	No collection/analysis	Charlson Comorbidity Index	Describe patient needs, symptoms, burden/experience
Barrio-Cortes *et al.* [39]	Spain	Cross-sectional	Community/primary care	2943	n/a	90.1	Collected only	Adjusted Morbidity Groups	Service utilisation or healthcare outcomes
Beach *et al.* [40]	USA	Prospective cohort/longitudinal	National datasets/registries	1564	1087	n/a	Collected only	Multiple Chronic Conditions	Describe patient needs, symptoms, burden/experience
Bekker *et al.* [41]	Netherlands	Retrospective cohort/record-based	Community/primary care	36	18	79^d^	No collection/analysis	None identified	Advance care planning (ACP)
Bernabeu-Wittel *et al.* [42]	Spain	Cross-sectional	Inpatient hospital	196	69	71.3	No collection/analysis	Charlson Comorbidity Index	Service utilisation or healthcare outcomes
Boyd *et al.* [43]	New Zealand	Prospective cohort/longitudinal	Nursing homes/long term care	256	152	n/a	Collected only	None identified	Describe patient needs, symptoms, burden/experience
Bushor and Rowser [44]	USA	Retrospective cohort/record-based	Outpatient/ambulatory care	24	0	82.3	Collected only	None identified	Evaluate palliative care models/interventions
Calsina-Berna *et al.* [45]	Spain	Cross-sectional	Inpatient hospital	236	143	68.2	No collection/analysis	Charlson Comorbidity Index	Describe patient needs, symptoms, burden/experience
Campos-Calderón *et al.* [46]	Spain	Retrospective cohort/record-based	Inpatient hospital	202	94	72.8	No collection/analysis	Charlson Comorbidity Index	Service utilisation or healthcare outcomes
Castany *et al.* [47]	Spain	Cross-sectional	Mixed settings	43	n/a	n/a	No collection/analysis	None identified	Service utilisation or healthcare outcomes
Cenzer *et al.* [48]	USA	Prospective cohort/longitudinal	National datasets/registries	8976	4927	81.3	Collected only	Simple disease count	Describe patient needs, symptoms, burden/experience
Chan and Pang [49]	Hong Kong	Cross-sectional	Nursing homes/long term care	287	224	83.8	No collection/analysis	Cumulative Illness Rating Scale	Quality of life or other patient-reported outcomes
Chen *et al.* [50]	Taiwan	Cross-sectional	National datasets/registries	505 569	n/a	n/a	No collection/analysis	Charlson Comorbidity Index	Service utilisation or healthcare outcomes
Chen *et al.* [51]	China	Retrospective cohort/record-based	Inpatient hospital	242	54	89^e^	No collection/analysis	Cumulative Illness Rating Scale	Service utilisation or healthcare outcomes
Chochinov *et al.* [52]	Canada	Cross-sectional	Mixed settings	404	194	74.2	No collection/analysis	Charlson Comorbidity Index	Describe patient needs, symptoms, burden/experience
Clarke *et al.* [53]	Canada	Qualitative	Community/primary care	35	19	79.5	No collection/analysis	Simple disease count	Describe patient needs, symptoms, burden/experience
Cummings *et al.* [54]	Canada	Mixed methods	Mixed settings	637	384	84.3	No collection/analysis	Simple disease count	Service utilisation or healthcare outcomes
Cunningham *et al.* [55]	UK ^f^	Qualitative	Community/primary care	4	2	75	No collection/analysis	Simple disease count	Describe patient needs, symptoms, burden/experience
Dahlqvist *et al.* [56]	Sweden	Retrospective cohort/record-based	Outpatient/ambulatory care	191	65	83.3	No collection/analysis	Simple disease count	Evaluate palliative care models/interventions
Disler *et al.* [57]	Australia	Retrospective cohort/record-based	Mixed settings	105	41	82^d^	No collection/analysis	Charlson Comorbidity Index	Access/referral to palliative care
Ebenau *et al.* [58]	Netherlands	Qualitative	Nursing homes/long term care	9	1	61	No collection/analysis	None identified	Access/referral to palliative care
Esteban-Burgos *et al.* [59]	Spain	Cross-sectional	Nursing homes/long term care	149	100	84.5	No collection/analysis	Simple disease count	Describe patient needs, symptoms, burden/experience
Etkind *et al.* [60]	UK	Qualitative	Hospice/specialist palliative care	15	8	80^e^	No collection/analysis	None identified	Describe patient needs, symptoms, burden/experience
Finucane *et al.* [61]	UK	Retrospective cohort/record-based	Nursing homes/long term care	77	n/a	n/a	No collection/analysis	None identified	Describe patient needs, symptoms, burden/experience
Finucane *et al.* [10]	UK	Retrospective cohort/record-based	National datasets/registries	15 725	8396	n/a	No collection/analysis	Simple disease count	Access/referral to palliative care
Francis *et al.* [62]	New Zealand	Qualitative	Community/primary care	14	8	n/a	Collected only	Simple disease count	Describe patient needs, symptoms, burden/experience
Frazer and Mobley [63]	USA	Mixed methods	Mixed settings	156	67	74.9	Collected only	Simple disease count	Quality of life or other patient-reported outcomes
Gill *et al.* [64]	Canada	Qualitative	Community/primary care	27	15	82.3	No collection/analysis	Simple disease count	Describe patient needs, symptoms, burden/experience
Ginggeaw *et al.* [65]	USA	Secondary analyses	National datasets/registries	245	137	n/a	Collected only	Simple disease count	Quality of life or other patient-reported outcomes
Gothe *et al.* [66]	Germany	Retrospective cohort/record-based	National datasets/registries	13 883	7654	n/a	Collected only	Elixhauser Comorbidity Measure	Access/referral to palliative care
Greenle *et al.* [67]	USA	Retrospective cohort/record-based	Community/primary care	126	65	84.8	No collection/analysis	Simple disease count	Service utilisation or healthcare outcomes
Haines *et al.* [68]	USA	Secondary analyses	National datasets/registries	656	n/a	n/a	Collected + discussed	Simple disease count	Service utilisation or healthcare outcomes
Harrison *et al.* [69]	USA	Secondary analyses	Hospice/specialist palliative care	715	n/a	n/a	Collected only	Simple disease count	Service utilisation or healthcare outcomes
Hash *et al.* [70]	USA	Cross-sectional	Community/primary care	102	n/a	69.2	Collected + discussed	Simple disease count	Advance care planning (ACP)
Holley *et al.* [71]	USA	Mixed methods	Community/primary care	20	n/a	n/a	Collected + discussed	Simple disease count	Evaluate palliative care models/interventions
Hung *et al.* [72]	Taiwan	Retrospective cohort/record-based	Inpatient hospital	20	n/a	n/a	No collection/analysis	None identified	Evaluate palliative care models/interventions
Kang *et al.* [73]	Taiwan	Secondary analyses	National datasets/registries	475	n/a	n/a	No collection/analysis	Charlson Comorbidity Index	Service utilisation or healthcare outcomes
Kasdorf *et al.* [74]	Germany	Cross-sectional	Mixed settings	213	n/a	n/a	Collected only	None identified	Access/referral to palliative care
Kato *et al.* [75]	Japan	Prospective cohort/longitudinal	Community/primary care	2142	844	n/a	No collection/analysis	Simple disease count	Describe patient needs, symptoms, burden/experience
Kramer and Auer [76]	USA	Mixed methods	Community/primary care	n/a	n/a	n/a	Collected + discussed	Simple disease count	Describe patient needs, symptoms, burden/experience
Kramer [77]	USA	Mixed methods	Community/primary care	14	7	n/a	Collected + discussed	Simple disease count	Describe patient needs, symptoms, burden/experience
Kricke *et al.* [78]	USA	Secondary analyses	National datasets/registries	477	264	n/a	Collected only	Simple disease count	Service utilisation or healthcare outcomes
Lee *et al.* [79]	Singapore	Qualitative	Mixed settings	0	0	n/a	No collection/analysis	None identified	Caregiver outcomes
Llop-Medina *et al.* [80]	Spain	Qualitative	Community/primary care	12	4	77.4	Collected only	None identified	Access/referral to palliative care
Luk *et al.* [81]	Hong Kong	Prospective cohort/longitudinal	Nursing homes/long term care	312	241	88	No collection/analysis	Simple disease count	Service utilisation or healthcare outcomes
Luo *et al.* [82]	Mixed >3	Secondary analyses	National datasets/registries	4532	n/a	n/a	No collection/analysis	Simple disease count	Service utilisation or healthcare outcomes
Maloney *et al.* [83]	USA	Secondary analyses	Mixed settings	122	n/a	n/a	Collected only	Simple disease count	Describe patient needs, symptoms, burden/experience
Marcucci *et al.* [84]	Brazil	Cross-sectional	Community/primary care	5	n/a	n/a	No collection/analysis	None identified	Access/referral to palliative care
Marventano *et al.* [85]	Spain	Cross-sectional	Nursing homes/long term care	816	699	84.8	No collection/analysis	Simple disease count	Describe patient needs, symptoms, burden/experience
Mason *et al.* [86]	UK	Qualitative	Mixed settings	19	9	72.8	No collection/analysis	None identified	Service utilisation or healthcare outcomes
Mason *et al.* [87]	UK	Qualitative	Mixed settings	37	14	76	No collection/analysis	None identified	Describe patient needs, symptoms, burden/experience
McDermott *et al.* [88]	USA	Secondary analyses	Mixed settings	2365	n/a	n/a	Collected only	Simple disease count	Service utilisation or healthcare outcomes
McGhan *et al.* [89]	USA	Qualitative	Outpatient/ambulatory care	1	n/a	n/a	Collected only	None identified	Caregiver outcomes
McKittrick *et al.* [90]	Australia	Secondary analyses	Community/primary care	89 266	n/a	82	Collected + discussed	Simple disease count	Describe patient needs, symptoms, burden/experience
McParland *et al.* [91]	UK	Qualitative	Emergency department	6	n/a	n/a	Collected only	None identified	Describe patient needs, symptoms, burden/experience
Méndez-Bailón *et al.* [92]	Spain	Cross-sectional	Inpatient hospital	3100	n/a	77.5	No collection/analysis	Charlson Comorbidity Index	Describe patient needs, symptoms, burden/experience
Mota-Romero *et al.* [93]	Spain	Cross-sectional	Nursing homes/long term care	149	100	84.5	No collection/analysis	Charlson Comorbidity Index	Access/referral to palliative care
Murali *et al.* [94]	USA & Australia	Secondary analyses	Hospice/specialist palliative care	213	n/a	n/a	Collected only	Charlson Comorbidity Index	Describe patient needs, symptoms, burden/experience
Murali *et al.* [95]	USA & Australia	Secondary analyses	Hospice/specialist palliative care	0	0	n/a	Collected only	Charlson Comorbidity Index	Describe patient needs, symptoms, burden/experience
Murali *et al.* [96]	USA & Australia	Secondary analyses	Hospice/specialist palliative care	296	n/a	n/a	Collected only	Charlson Comorbidity Index	Describe patient needs, symptoms, burden/experience
Ng *et al.* [97]	Canada	Cross-sectional	Nursing homes/long term care	1774	n/a	n/a	No collection/analysis	Simple disease count	Service utilisation or healthcare outcomes
Nicholson *et al.* [98]	UK	Cross-sectional	Community/primary care	815	517	88^e^	Collected only	None identified	Access/referral to palliative care
Nkhoma *et al.* [99]	Ghana; Malawi; Zimbabwe	Qualitative	Community/primary care	41	27	62.3	No collection/analysis	None identified	Evaluate palliative care models/interventions
Nothelle *et al.* [100]	USA	Retrospective cohort/record-based	National datasets/registries	560	n/a	n/a	Collected + discussed	Charlson Comorbidity Index	Service utilisation or healthcare outcomes
Parker *et al.* [101]	Australia	Prospective cohort/longitudinal	Nursing homes/long term care	69	47	84	No collection/analysis	Simple disease count	Access/referral to palliative care
Piers *et al.* [102]	Belgium	Prospective cohort/longitudinal	Inpatient hospital	70	n/a	n/a	No collection/analysis	Charlson Comorbidity Index	Service utilisation or healthcare outcomes
Pop *et al.* [103]	Romania	Prospective cohort/longitudinal	Inpatient hospital	72	n/a	78.4	No collection/analysis	Simple disease count	Caregiver outcomes
Portz *et al.* [104]	USA	Secondary analyses	Community/primary care	381	171	74.1	No collection/analysis	Charlson Comorbidity Index	Describe patient needs, symptoms, burden/experience
Reinke *et al.* [105]	USA	Cross-sectional	Community/primary care	503	4	71.1	Collected only	Elixhauser Comorbidity Measure	Describe patient needs, symptoms, burden/experience
Reis Júnior *et al.* [106]	Brazil	Cross-sectional	Community/primary care	11 177	6617	69.8	No collection/analysis	Simple disease count	Describe patient needs, symptoms, burden/experience
Robinson *et al.* [107]	Australia	Prospective cohort/longitudinal	Community/primary care	52	28	76.6	No collection/analysis	Simple disease count	Quality of life or other patient-reported outcomes
Rodríguez-Álvaro *et al.* [108]	Spain	Cross-sectional	Community/primary care	978	n/a	80	No collection/analysis	Charlson Comorbidity Index	Describe patient needs, symptoms, burden/experience
Romanò *et al.* [109]	Italy	Retrospective cohort/record-based	Community/primary care	224	n/a	83.5	No collection/analysis	Simple disease count	Describe patient needs, symptoms, burden/experience
Rosella *et al.* [110]	Canada	Retrospective cohort/record-based	Community/primary care	64 753	n/a	n/a	Collected only	Simple disease count	Service utilisation or healthcare outcomes
Safabakhsh *et al.* [111]	Iran	Retrospective cohort/record-based	Inpatient hospital	44 167	n/a	n/a	No collection/analysis	Simple disease count	Access/referral to palliative care
Salinas *et al.* [112]	Mexico	Cross-sectional	Inpatient hospital	50	29	58	No collection/analysis	Simple disease count	Describe patient needs, symptoms, burden/experience
Santaeugènia *et al.* [113]	Spain	Retrospective cohort/record-based	Community/primary care	401 944	222 483	n/a	No collection/analysis	Simple disease count	Service utilisation or healthcare outcomes
Schellinger *et al.* [114]	USA	Cross-sectional	Community/primary care	160	77	79	No collection/analysis	None identified	Advance care planning (ACP)
Skilbeck *et al.* [115]	UK	Qualitative	Community/primary care	10	7	84^e^	No collection/analysis	None identified	Describe patient needs, symptoms, burden/experience
Sri-on *et al.* [116]	Thailand	Retrospective cohort/record-based	Inpatient hospital	161	79	79.8	No collection/analysis	Charlson Comorbidity Index	Access/referral to palliative care
Suriyawongpaisal *et al.* [117]	Thailand	Cross-sectional	Community/primary care	1686	n/a	n/a	No collection/analysis	None identified	Evaluate palliative care models/interventions
Tagliafico *et al.* [118]	Italy	Cross-sectional	Mixed settings	422	n/a	82.8	No collection/analysis	Cumulative Illness Rating Scale	Access/referral to palliative care
Tan *et al.* [119]	Australia	Retrospective cohort/record-based	Emergency department	55	28	84	No collection/analysis	None identified	Describe patient needs, symptoms, burden/experience
Tanuseputro *et al.* [120]	Canada	Retrospective cohort/record-based	Community/primary care	177 817	n/a	n/a	No collection/analysis	Simple disease count	Access/referral to palliative care
Tschirhart *et al.* [121]	USA	Cross-sectional	Emergency department	73 494	n/a	83.2	Collected + discussed	Elixhauser Comorbidity Measure	Service utilisation or healthcare outcomes
Wagner *et al.* [122]	USA	Retrospective cohort/record-based	Community/primary care	10 899	4203	65.8	No collection/analysis	Simple disease count	Service utilisation or healthcare outcomes
Wu *et al.* [123]	Taiwan	Prospective cohort/longitudinal	Community/primary care	41	n/a	67.6	No collection/analysis	None identified	Caregiver outcomes
Wu-Jun *et al.* [124]	China	Mixed methods	Inpatient hospital	3	3	63.9	No collection/analysis	Charlson Comorbidity Index	Evaluate palliative care models/interventions

^a^See *Appendix 5* for ungrouped values

^b^Other than descriptive/covariant, or to note as study limitation

^c^United States of America

^d^Age of death

^e^Median age (mean age not available)

^f^United Kingdom.

**Table 2:** Key data charts from the included studies.

Publication year (*n* = 90)	n	%
2005–9	3	3.3%
2010–14	13	14.4%
2015–19	24	26.7%
2020–24	33	36.7%
2025	17	18.9%
**Geographic location of study** (*n* = 90)	**n**	**%**
USA	20	22.2%
Spain	14	15.6%
UK	10	11.1%
Canada	8	8.9%
Australia	6	6.7%
Taiwan	3	3.3%
Netherlands	2	2.2%
Germany	2	2.2%
Italy	2	2.2%
Hong Kong	2	2.2%
New Zealand	2	2.2%
China	2	2.2%
Brazil	2	2.2%
Thailand	2	2.2%
Mixed/multi-country (>3 countries)	1	1.1%
Jordan	1	1.1%
Iran	1	1.1%
Japan	1	1.1%
Romania	1	1.1%
Singapore	1	1.1%
Sweden	1	1.1%
Belgium	1	1.1%
Mexico	1	1.1%
Ghana/Malawi/Zimbabwe (multi-country)	1	1.1%
**World Bank Income category** (2024/2025) (*n* = 90)	**n**	**%**
High-income country (HIC)	84	93.3%
Upper-middle-income country (UMIC)	4	4.4%
Low/lower-middle-income country (LMIC/LIC)	2	2.2%
**MLTC participant numbers (by study sample)** (*n* = 90)^a^	**n**	**%**
< 50	22	24.4%
50–199	16	17.8%
200–999	22	24.4%
1000–9999	13	14.4%
≥ 10 000	9	10.0%
Not stated/not applicable	8	8.9%
**Mean ages of MLTC participants** (*n* = 52)^b^	**n**	**%**
< 60 years	1	1.9%
60–69 years	8	15.4%
70–79 years	18	34.6%
80–89 years	24	46.2%
≥ 90 years	1	1.9%
**Sex distributions of MLTC participants** (*n* = 53)^c^	**n**	**%**
Male	215,814	45.1%
Female	262,488	54.9%
**Study-level sex predominance** (*n* = 53)^d^	**n**	**%**
Female majority (>50% female)	30	56.6%
Male majority (>50% male)	20	37.7%
Equal (50% male/50% female)	3	5.7%
**Race/ethnicity data collection and analysis** (*n* = 90)^e^	**n**	**%**
No collection or analysis	55	61.1%
Collected only (not analysed)	27	30.0%
Collected and discussed	8	8.9%
**Study designs** (*n* = 90)	**n**	**%**
Cross-sectional	23	25.6%
Retrospective cohort/record-based	22	24.4%
Qualitative	17	18.9%
Secondary analyses	12	13.3%
Prospective cohort/longitudinal	11	12.2%
Mixed methods	5	5.6%
**Study settings** (*n* = 90)	**n**	**%**
Community/primary care	22	24.4%
Inpatient hospital	17	18.9%
National datasets/registries	16	17.8%
Mixed settings	14	15.6%
Nursing homes/long-term care	10	11.1%
Hospice/specialist palliative care	5	5.6%
Outpatient/ambulatory care	3	3.3%
Emergency department	3	3.3%
**Study primary aims** (*n* = 90)	**n**	**%**
Describe patient needs, symptoms, burden/experience	34	37.8%
Service utilisation or healthcare outcomes	24	26.7%
Access/referral to palliative care	14	15.6%
Evaluate palliative care models/interventions (needs reported as secondary aim)	7	7.8%
Quality of life or other patient-reported outcomes	4	4.4%
Caregiver outcomes	4	4.4%
Advance care planning (ACP)	3	3.3%

^a^Data presented here are of groupings of the sample size (number of eligible MLTC patients)

^b^Distribution of reported mean/median ages (reported in *n* = 52 studies, *n* = 38 studies not reported or not able to extrapolate MLTC population data)

^c^Pooled sex distribution across studies reporting sex (*n* = 53 studies, *n* = 37 studies not reported or not able to extrapolate MLTC population data)

^d^Across the studies that reported the sex of participants that could be disaggregated from the wider study dataset (*n* studies = 53, *n* participants = 478 302), men made up 45.1% of study participants)

^e^Collected and discussed refers to studies that collected and discussed race/ethnicity other than descriptive/covariant data, or when noted as a study limitation

Across the studies, the total number of eligible participants with MLTC was 1 455 036. Of the studies that reported the gender of participants that could be disaggregated from the wider study dataset (*n* studies = 53, *n* participants = 478 302), men made up 45.1% of study participants. Only eight studies (9%) collected and discussed race/ethnicity other than descriptive/covariant data, or when noted as a study limitation.

#### Study design, setting and aims

Most studies used quantitative designs (*n* = 68, 75.6%), with 17 qualitative studies (18.9%) and five using mixed methods (5.6%). Among the quantitative studies, cross-sectional, retrospective cohort, secondary analysis and prospective cohort designs were represented in broadly similar numbers. Community/primary care, inpatient hospitals and national datasets were the most common study settings. There were relatively low numbers of studies set in hospice/specialist palliative care settings (*n* = 5); these were conducted in the USA (*n* = 1), the USA and Australia (*n* = 3), and UK (*n* = 1). The aims of each study were assigned to a single best-fit category, with the most prevalent category being ‘Describe patient needs, symptoms, burden/experience’ (*n* = 34, 37.8%), followed by ‘Service utilisation or healthcare outcomes’ (*n* = 24, 26.7%). Together, those two accounted for nearly two-thirds of all studies; the remaining five categories each had 14 or fewer studies.

#### Definitions of multiple long-term conditions

MLTC was most commonly operationalised as a simple count threshold (e.g. ≥2 or ≥3 long-term conditions) in 42 studies (46.7%), or via a comorbidity index/tool in 34 studies (37.8%). The most frequently used comorbidity index/tool was the Charlson Comorbidity Index (*n* = 24, 26.7%) and less often the Cumulative Illness Rating Scale (*n* = 3, 3.3%) or the Elixhauser Comorbidity Measure (*n* = 3, 3.3%). Thirty-one studies (34.4%) used versions of the World Health Organization (WHO) International Statistical Classification of Diseases and Related Health Problems (ICD) to code health domains [125].

### Mapping overview: domains of need

Fifteen subdomains of palliative care needs were identified. Physical needs were the most frequently reported domain, documented in 72 studies (80.0%). A further three domains (functional needs, prognostic uncertainty and reactive needs, and sociodemographic inequality) were each reported in 69 studies (76.7%), followed by psychological and coordination needs in 65 studies (72.2%). In total, six of the 15 domains were addressed by at least 70% of the evidence base. Coverage across studies was heterogeneous: the median number of domains addressed per study was nine (mean 8.7). Only two studies [58, 116] reported across all 15 domains, while 16 studies (17.8%) addressed four or fewer.

To address this heterogenous coverage, the 15 subdomains were placed into four overarching domains that aligned the review’s findings with established conceptual frameworks in geriatrics and palliative care for older adults with MLTC (Table 3). Quantitative mapping of the domains and subdomains is detailed in Appendix 6; ungrouped values for design, setting and study aim are given in Appendix 5.

**Table 3:** Selection of the study’s four main domains.

Main domain	Conceptual frameworks	Grouping	Rationale	Need (sub-domain)	Studies (*n* = 90)	% of studies	Domain coverage
**Comprehensive support needs in the context of functional decline**	Comprehensive Geriatric Assessment [126] Frailty frameworks [127]	Functional, physical, psychological, spiritual, social, financial and family/carer needs.	Grouping these sub-domains together reflects the dominant conceptualisation of frailty and multimorbidity as inherently multidimensional rather than purely biomedical [128].	Need for functional support Need for physical symptom management Need for psychological support Need for spiritual care Need for social support Need for financial support Need for family/carer support	69 72 65 23 52 21 51	76.7% 80.0% 72.2% 25.6% 57.8% 23.3% 56.7%	56.0%
**Proactive care needs shaped by limited death literacy and prognostic uncertainty**	Advance care planning [129] Death literacy [130] Prognostic uncertainty [131]	Advance care planning (ACP), goal-concordant care, and prognostic uncertainty/episodic deterioration/reactive care.	As the trajectory of MLTC is characteristically non-linear, prognostic ambiguity creates the conditions under which ACP and goal-concordant care become both more necessary and more difficult [132, 133].	Need for advance care planning Need for goal-concordant care Need to manage prognostic uncertainty/episodic deterioration/reactive care	39 32 69	43.3% 35.6% 76.7%	51.9%
**Systems-level needs for coordination and collaboration**	Integrated Care Framework [134]	Communication needs, coordination needs, and Multidisciplinary Team (MDT)/collaboration needs.	Older adults with MLTC interact with multiple providers, sectors, and care settings, and the resulting discontinuity is repeatedly identified as a primary driver of unmet need [135].	Need for effective communication Need for coordination across services Need for multidisciplinary collaboration	59 65 54	65.6% 72.2% 60.0%	65.9%
**Compounded needs arising from inequality**	Social Determinants of Health Framework [136, 137]	Diagnostic inequality and sociodemographic inequality.	There is strong, recent evidence that socioeconomic position, ethnicity, sex, and related axes of disadvantage shape both the incidence and trajectory of frailty and MLTC, and that these social determinants act over the life course [138].	Need to address diagnostic inequality Need to address sociodemographic inequality	46 69	51.1% 76.7%	63.9%

### Narrative synthesis by domain

#### Comprehensive support needs in the context of functional decline

‘Physical symptom management needs’ were common across settings (72/90, 80.0%). Pain, dyspnoea, fatigue and neuropsychiatric symptoms often co-occurred [96, 104]. Terminal hospital admissions for congestive heart failure, chronic obstructive pulmonary disease and end-stage renal failure showed limited routine palliative care despite heavy symptom burden [57]. In long-term care symptom load remained high, with many residents dying with unaddressed needs [43].

‘Functional support needs’ were highly prevalent (69/90, 76.7%). Large cohort analyses described substantial functional limitation and downstream consequences of unmet support needs, including loss of independence and rising care dependency [40, 100]. In home care cohorts, participants commonly had high MLTC burden with progressive decline, requiring increasingly complex packages of support [67, 107]. Recent longitudinal evidence also links complex MLTC to subsequent disability trajectories [75], with immobility, activities of daily living dependence and limited access to rehabilitation particularly evident among frail older adults and where neurodegenerative conditions were present [39, 81, 85].

‘Psychological support needs’*,* including anxiety, depression and dignity related distress, were frequently reported (65/90, 72.2%). Loneliness and social isolation were independently associated with worse end of life experiences in people with MLTC [36]. Ebenau *et al.* [58] described profound existential distress, including suicidal ideation, among patients with MLTC and substance use histories, while Hash *et al.* [70] and Gill *et al.* [64] linked emotional burden to illness complexity and caregiver strain.

‘Spiritual care needs’ were reported less frequently (23/90, 25.6%) but were important when explored. Patients described needs for meaning making, inner peace and culturally sensitive support, with barriers including language, diverse religious and cultural practices that services were not equipped to accommodate, and limited training among staff in the inpatient hospital and long-term care settings in which these studies were conducted [38, 49, 58].

‘Social and financial support needs’ were closely intertwined. Social support needs (52/90, 57.8%) were commonly framed through practical and relational dependencies, with heavy reliance on informal carers, typically spouses or adult children, often at significant personal cost to the carer [79, 89]. Where informal care networks were insufficient, the use of formal care packages escalated as functional decline progressed [76, 107], and decisions about long term care admission frequently arose when informal support networks could no longer sustain safety or quality of life [43, 113]. Financial support needs (21/90, 23.3%) were described in relation to out of pocket costs for home adaptations, paid carers and transport, and navigating complex state benefits systems, with disproportionate impacts on people on low incomes and those outside cancer-centric pathways [77, 110].

‘Family and carer support needs’ were common (51/90, 56.7%) and reflected sustained physical and emotional workload. Informal carers experienced substantial practical burden and psychological strain, often with limited respite support [79, 89]. Burden was frequently greater in non-malignant trajectories, characterised by prolonged dependency and delayed access to palliative services [103, 123]. Gendered impacts were evident, with disproportionate burden on women caregivers who were less likely to receive financial and respite support [79, 84].

#### Proactive care needs shaped by limited death literacy and prognostic uncertainty

Managing prognostic uncertainty, episodic deterioration and reactive care response were identified as major barriers to timely recognition of the palliative phase**.** These barriers often resulted in reactive rather than anticipatory care, particularly in generalist settings where decline was difficult to identify and trajectories were unpredictable [87, 121]. In emergency and acute care, missed opportunities to identify palliative need were documented [116], and clinicians reported difficulty aligning escalation decisions with limited prognosis [119]. Patients and families were frequently excluded from meaningful discussions, contributing to misaligned care and late transitions [64, 116]. Contemporary multinational evidence also showed that higher condition burden is associated with increased risk of escalation and prolonged acute care use near the end of life, reinforcing the importance of earlier identification and planning [82].

‘Advance care planning (ACP) needs’ were evident across all illness trajectories but were often greater in non-malignant MLTC. When people had MLTC and cancer, ACP typically occurred earlier and was more detailed, whereas in non-malignant MLTC it was more commonly delayed and crisis-driven [41, 76]. This was attributed to limited identification of decline or a palliative phase unless an acute crisis occurred, meaning planning was reactive rather than proactive [41]. Where ACP was present, this was associated with lower acute care utilisation and greater alignment with patient preferences [68, 78, 88]. Community-based qualitative work showed many older adults with MLTC engaged in anticipatory thinking but lacked support to translate wishes into actionable plans [53, 62].

‘Goal concordant care needs’ reflected ongoing challenges in aligning treatment and escalation decisions with patient priorities across settings. Even when preferences were discussed, prognostic uncertainty, fragmented care and weak communication across services could undermine their implementation, contributing to unwanted interventions and delayed transitions [64].

#### Systems-level needs for coordination and collaboration

‘The need for coordination across services’ was commonly reported (65/90, 72.2%) and was characterised by fragmented pathways, duplication and unclear accountability. Problems were most visible at transition points, including between long-term care and emergency departments [54], admission to rehabilitation units [119], and hospital admissions and discharge [57]. Community and primary care exemplars highlighted the value of care coordination roles and shared records for older adults with MLTC and frailty [98], while systems level programmes using population stratification identified large cohorts with advanced need but variable access to palliative input [113]. Integrated care approaches were associated with improved continuity and fewer hospitalisations, but availability was inconsistent [67, 119].

‘Multidisciplinary collaboration needs’ were evident in over half of studies (54/90, 60.0%). Capacity to benefit from integrated models involving nurses, allied health professionals and physicians was highlighted, particularly in relation to service navigation and continuity provision for people with complex MLTC [76, 77]. However, many studies reported late or absent specialist palliative care involvement, leaving generalists to manage substantial complexity without consistent specialist support [109, 118].

‘Effective communication needs’ were widely reported (59/90, 65.6%) and underpinned both coordination and collaborative working. Studies described gaps in shared decision making and information exchange across care settings, contributing to inconsistent plans and repeated distressing conversations [54, 77, 110]. Recent co-design work emphasised the role of structured communication processes and improved documentation to support person centred planning across services [60], while ethnographic data from emergency care illustrated how communication breakdowns can contribute to disengagement and missed opportunities for alignment with patient priorities [91].

#### Compounded needs arising from inequality

‘Diagnostic inequalities’ were frequently reported (46/90, 51.1%), with people living with non-cancer MLTC less likely to be recognised as having palliative needs or to access hospice and specialist palliative care. Studies consistently described later identification and fewer referrals for non-cancer trajectories compared with cancer [66, 73]. Record-based analyses also indicated later referral patterns and higher acute care utilisation for people with MLTC, consistent with reliance on crisis triggers rather than earlier needs-based identification [120, 122].

‘Sociodemographic inequalities’ were common (69/90, 76.7%) and reflected disparities by income, geography and ethnicity in access, utilisation, place of care and caregiver burden. Studies reported unequal access to coordinated services and higher out of pocket costs among disadvantaged groups, contributing to unmet needs and strain on families [76, 110]. Evidence from LMIC settings highlights similar patterns, with poverty, travel costs, limited service availability and underdeveloped palliative care infrastructure amplifying unmet need [99, 106, 117]. Intersecting social adversity and stigma further compounded need in some populations. Studies highlighted barriers to access and fragmented care for people facing complex health and social challenges, including those with substance use disorders, where stigma and exclusion shaped both service engagement and the experience of distress [58]. Only eight studies (8.9%) collected and discussed race/ethnicity other than descriptive/covariant data or when noted as a study limitation, reinforcing concerns about equality in palliative care for people with MLTC. [139].

### Summary of patterns across domains

The need for comprehensive, anticipatory care was evident across all domains. Functional decline was described as the final common pathway in advanced MLTC, accompanied by high symptom burden and psychosocial distress. Addressing prognostic uncertainty and initiating timely planning remained critical yet often unmet, particularly in non-malignant trajectories, where reactive, crisis-led responses predominated. Persistent gaps in system integration and multidisciplinary collaboration compounded these challenges, while diagnostic and sociodemographic inequalities consistently delayed recognition and access to appropriate support. Support for caregivers was acknowledged but rarely measured in depth, leaving families to shoulder sustained physical and emotional strain with limited formal assistance. Heterogeneity in populations, definitions and outcome reporting meant that no consistent temporal or subgroup patterns in reported needs could be discerned.

## Discussion

This scoping review of 90 studies highlights persistent unmet needs for palliative care among people with MLTC. These needs are often overlooked within systems oriented toward life-prolonging or single-disease models, despite international policy emphasising the importance of responding to the complexity of ageing populations [140].

From the review findings, a four-domain structure of need was identified: (i) Functional support, (ii) Anticipatory care, (iii) Integrated systems and (iv) Equity of access. This structure aligns broadly with findings from thematically adjacent reviews. Nicholson *et al.* [141], in a scoping review of community-dwelling older people with multimorbidity, identified the most prevalent palliative care needs as pain, function, future planning and maintaining social connection, aligned with our first two domains. The review by Stow *et al.* [18] of end-of-life care needs in frailty, similarly found that functional support needs were high and that individuals’ preferences for reduced intervention were not consistently observed. Llop-Medina *et al.* [80] documented a variety of unaddressed palliative care needs among older patients with multimorbidities, alongside complexity in detecting those needs and identifying referral pathways. The current review builds upon the literature in its explicit identification of inequality as a domain of need in its own right, and by highlighting the importance of systems-level coordination.

### Functional support

Functional support was the need most frequently reported across the included studies (69/90, 76.7%). However, because only those needs that were directly assessed could be reported, this reflects the focus of the evidence base rather than the prevalence or severity of need. In studies that examined patient priorities, loss of independence in daily activities was prioritised by older adults over disease-specific outcomes, reflecting the cumulative effect of deterioration across physical, cognitive, emotional and social domains [142, 143]. This decline was experienced not only as disability but also as loss of dignity and identity, reinforcing practical functional support as a core palliative priority. Evidence from rehabilitation-focused palliative care demonstrates that personalised interventions can improve activity and participation, even in advanced illness [143]. International frameworks, including WHO’s Integrated Care for Older People and National Institute for Health and Care Excellence guidance, emphasise multidisciplinary approaches to preserve function and autonomy [144, 145].

### Anticipatory care

The need for proactive, person-centred planning was evident across all trajectories but was comparatively greater in non-malignant MLTC. While cancer care typically involved earlier and more detailed ACP, the journeys of people with non-malignant MLTC were characterised by delayed and crisis-driven discussions [6, 41, 146]. Structured ACP interventions improve quality of life and reduce burdensome interventions [147, 148], yet their implementation outside oncology remains limited.

### Integrated systems

The need for integrated, coordinated systems was a recurring theme. People with MLTC frequently navigate fragmented care with poor continuity, unclear accountability and weak links between primary, specialist and social care. Such fragmentation increases vulnerability and often results in avoidable hospitalisation or late-stage crisis management [19, 149]. Policy frameworks, including the UK’s Ambitions for Palliative and End of Life Care and WHO’s Decade of Healthy Ageing, emphasise shared records and multidisciplinary collaboration as priorities [150, 151].

### Equity of access

The need to address inequalities arising from diagnostic exclusion and structural disadvantage was clear. People with non-malignant MLTC were less likely to have palliative needs identified than those with cancer, reflecting persistent single-disease models of care [152]. These diagnostic gaps were compounded by social inequalities across socioeconomic status and ethnicity. International evidence confirms that marginalised groups experience worse outcomes due to cultural misunderstanding, limited resources and systemic bias [153, 154]. Embedding equity through culturally adapted interventions and inclusive pathways is essential to person-centred palliative care for people with MLTC.

### Implications

The review identified several key evidence gaps. The limited reporting of financial and spiritual needs, identified in only 23.3% and 25.6% of studies respectively, is notable in a population for whom care is often prolonged, costly and existentially demanding. Both domains should arguably be considered essential components of comprehensive needs assessment for older adults with MLTC. Goal-concordant care needs were reported in only around one third of included studies (35.6%), with studies more often documenting service provision than whether care was aligned with patient preferences. Inequality was highlighted in the majority of studies, including sociodemographic inequality in 69/90 studies (76.7%) and diagnostic inequality in 46/90 studies (51.1%). However, most studies identified inequity as a problem rather than explaining how access to palliative care could be made more equitable for people with MLTC. The need for caregiver support was acknowledged but rarely measured in depth. Informal carers sustain care yet often lack resources, facing sustained physical and emotional strain. Comparative studies indicate that burden is greater for non-cancer MLTC than cancer, driven by longer trajectories, higher dependency and delayed access to services [49, 103]. Dyadic interventions show promise in reducing caregiver burden and improving quality of life [155], but evidence remains limited and concentrated in high-income settings.

These evidence gaps were integrated into the synthesis of the review findings, from which were generated the implications for practice and future research summarised in Table 4.

**Table 4:** Implications for practice and research.

**Theme**	**Practice implications**	**Enablers**	**Future research priorities**
**Functional support**	Make independence/autonomy core outcomes; embed Allied Health Professionals (AHPs) in routine multidisciplinary team (MDT) care; set function-focused goals and reviews.	Workforce upskilling in rehab-focused palliative care; protected coordination time; caregiver training/respite; interoperable records to track function over time.	Trials of AHP-led, function-first models; patient-reported outcomes on function/autonomy/participation; implementation fidelity and scalability; greater attention to the needs and support roles of family and carers.
**Anticipatory care**	Promote preparedness via public engagement and staff training; deliver ACP as a flexible, revisitable process under uncertainty, with clear triggers to review (e.g. step-changes in function, new diagnosis/exacerbation, discharge/transition, carer strain).	Communication & prognostication training; Electronic Health Record (EHR) prompts/templates to normalise revisiting plans; inclusion of caregivers; community engagement resources.	Evaluate flexible, re-visitable ACP; decision aids; acceptability (patients/families/clinicians) and reach; equity (incl. underserved groups); trigger strategies (which events/thresholds prompt ACP review) and their effectiveness.
**Integrated systems**	Invest in digital infrastructure/shared records; define coordination roles; strengthen continuity, accountability, and MDT handovers.	Dedicated care-coordination roles/time; shared care plans across settings; data standards and interoperability; simple referral/navigation pathways.	Effectiveness of shared-record solutions; role-clarity models; continuity/accountability metrics; coordination measures (e.g. continuity indices, relational-coordination scales, care-plan completeness/handover timeliness); cost-effectiveness and sustainability.
**Equity of access**	Embed culturally competent, inclusive practice; co-design with communities; address structural barriers to access.	Cultural-competence training; community partnerships; tailored caregiver/navigation support; monitoring equity metrics in services and outcomes.	Address HIC predominance and limited LMIC applicability; equity-focused evaluations (esp. LMICs); culturally adapted models; stratified outcomes; community-partnered designs.

## Limitations

This review followed a scoping methodology, designed to map the breadth of existing literature rather than appraise study quality or synthesise effect sizes. This is consistent with our aims; however, the methodological characteristics of this approach should be taken into consideration when interpreting the findings. In particular, critical appraisal was not undertaken, and the frequency with which a need was reported identifies the focus and design of the primary literature rather than the prevalence, severity or relative importance of that need; comparisons of magnitude between domains should therefore not be drawn. Practical decisions, such as the use of age thresholds and database restrictions, were necessary to ensure feasibility but may have affected the generalisability and completeness of the review. Additionally, most included studies originated from high-income countries, reducing the global applicability of findings. This reflects a limitation of the current evidence base rather than the review methodology.

## Conclusion

Older adults with MLTC live with interdependent, and often unaddressed, palliative needs across four domains: (i) comprehensive support needs in the context of functional decline; (ii) proactive care needs shaped by limited death literacy and prognostic uncertainty; (iii) systems-level needs for coordination and collaboration; and (iv) compounded needs arising from inequality (diagnostic/sociodemographic). Functional decline was the most commonly described pathway through which needs manifest, and shaped the care priorities reported by people with MLTC and their families. Triggered, evolving ACP is needed to enable goal-concordant care, i.e currently lacking. Stronger coordination is also needed, delivered through effective multidisciplinary working and health and social care record-sharing. Equity must be explicit, through culturally competent practice and sustained community partnership.

## Supplementary Material

Supplementary_material_afag324

## Research Data Transparency and Availability

Search strategies and filters sufficient to replicate our searches are available as supplemental materials. All papers included in this review are referenced and available via their respective journals.
