# Supplementary material for: The palliative care needs of people with multiple long-term conditions and their family caregivers: a scoping review of mixed study designs
Source: Age Ageing. 2026 Nov 13;55(11):afag324. doi: 10.1093/ageing/afag324 (PMC13761345; doi:10.1093/ageing/afag324)
Supplement: Supplementary file 1 [file supplementary_material_afag324.zip › aa-26-1473-File004.docx]

The Palliative Care Needs of People with Multiple Long-Term Conditions (MLTC) and their Family Caregivers: A Scoping Review of Mixed Study Designs

## **Appendix 2: Deviations from the registered PROSPERO protocol (CRD42024510220)**

| **Protocol component** | **Protocol specification** | **Review as conducted** | **Rationale for deviation** |
| --- | --- | --- | --- |
| **Review type** | Systematic review with narrative synthesis | Scoping review of mixed study designs | During initial literature searches it became clear that the literature was highly heterogeneous across populations, definitions of MLTC, outcomes, settings, and study design. A scoping review approach was therefore adopted to allow structured mapping and synthesis of the evidence base, in line with JBI and PRISMA‑ScR guidance, rather than to appraise quality, or synthesise effects or outcomes. |
| **Critical appraisal** | Planned risk of bias assessment using JBI tools | No formal critical appraisal undertaken | Following adoption of a scoping review methodology, critical appraisal was not undertaken, consistent with scoping review standards that prioritise breadth, transparency, and identification of gaps over study quality assessment. Patterns, gaps, and limitations are instead described narratively across domains. |
| **Population: age criteria** | Adults ≥18 years with MLTC | Adults ≥50 years with MLTC | An age threshold was introduced to improve conceptual coherence and feasibility, reflecting that advanced MLTC and palliative trajectories predominantly affect older adults, while still capturing earlier onset associated with socioeconomic disadvantage (multimorbidity onset occurs 10 to 15 years earlier in the most deprived compared with the most affluent areas, and the median age of onset has fallen while socioeconomic inequalities have widened, particularly below 65 years; see Methods and Appendix 3 for the supporting references). This reduced heterogeneity arising from inclusion of early‑life or childhood‑onset conditions. |
| **Population: disease stage** | Broad inclusion of people “approaching end of life” | MLTC plus ≥1 indicator of a palliative phase (e.g. functional decline, limited prognosis) | Eligibility criteria were refined to ensure included studies explicitly addressed needs relevant to palliative and end‑of‑life care, rather than early‑stage multimorbidity. This strengthened alignment with the review objectives without altering the research question. |
| **Study types** | Original studies, review articles, and grey literature | Original empirical studies only (quantitative, qualitative, mixed methods) | Given the size and diversity of the peer‑reviewed empirical literature, inclusion was restricted to original studies to ensure analytic clarity and avoid duplication of findings from secondary syntheses. Conceptual and policy insights from reviews and grey literature were incorporated contextually in the discussion. |
| **Grey literature** | Planned search of UK grey literature sources | Grey literature not included | Inclusion of grey literature proved impractical within the scope of the review and added limited empirical data on needs trajectories. The review therefore prioritised peer‑reviewed international evidence, while retaining relevance to UK practice through interpretation and policy‑linked discussion. |
| **Search end date** | Review end date June 2024 | Searches extended to September 2025 | The search period was extended to capture a rapidly expanding and contemporary evidence base, particularly post‑COVID‑19. This enhanced completeness and relevance, without changing the review question or analytic framework. |
| **Data synthesis approach** | Narrative synthesis of outcomes | Inductive mapping of needs into domains and subdomains; theory‑informed synthesis | As data charting progressed, a more explicit conceptual framework was required to organise the breadth of needs identified. Established needs theory and MLTC/frailty frameworks were therefore applied analytically to strengthen interpretability and relevance for ageing and palliative care audiences. |
| **Geographical emphasis** | Explicit UK grey literature component | International empirical focus; UK relevance through interpretation | While the protocol included a UK‑specific grey literature element, the final review prioritised international empirical studies. UK policy and practice implications were addressed in the discussion, consistent with *Age & Ageing*’s international scope. |
