# Supplementary material for: The palliative care needs of people with multiple long-term conditions and their family caregivers: a scoping review of mixed study designs
Source: Age Ageing. 2026 Nov 13;55(11):afag324. doi: 10.1093/ageing/afag324 (PMC13761345; doi:10.1093/ageing/afag324)
Supplement: Supplementary file 1 [file supplementary_material_afag324.zip › aa-26-1473-File005.docx]

The Palliative Care Needs of People with Multiple Long-Term Conditions (MLTC) and their Family Caregivers: A Scoping Review of Mixed Study Designs

## **Appendix 3: Eligibility criteria**

Study eligibility criteria, including definition of the “palliative phase” for people with MLTC

| **Domain** | | **Criteria** | | **Description / Examples** | |
| --- | --- | --- | --- | --- | --- |
| **Population** | **Inclusion** | | Adults aged ≥50 years with MLTC^1^, defined as ≥2 long term conditions plus ≥1 additional “palliative phase” characteristic (see below). Adult paid/unpaid caregivers (≥18 years) of individuals with MLTC in the palliative phase. *Rationale:* Lower age cut-off captures individuals from socioeconomically deprived and ethnically diverse populations who experience earlier MLTC onset and premature mortality [1,2]. Excludes younger adults with lifelong conditions originating in childhood. | |  |
|  | **Exclusion** | | Early-stage MLTC without indicators of advanced disease, functional decline, or limited prognosis. Studies focusing solely on a single index condition. Non-disaggregated MLTC population data, studies in which adults aged ≥50 are not the majority MLTC population, or studies in which mean or median age of MLTC population is ≤49. | |  |
| **Concept** | **Inclusion** | | Multidimensional palliative and/or symptomatic needs (physical, psychological, social, spiritual) in individuals or caregivers.  People with MLTC or caregivers making provision for death and dying. | |  |
|  | **Exclusion** | | Absence of need-related data. Focus solely on disease-modifying or curative treatment. | |  |
| **Context** | **Inclusion** | | All care settings and countries; all models of care.  Original empirical research (quantitative, qualitative, or mixed methods). | |  |
|  | **Exclusion** | | Abstracts, protocols, non-original research (e.g. reviews, editorials, opinion pieces). | |  |
| **Palliative Phase Characteristics**  (at least one required in addition to ≥2 long-term conditions) | **Diagnosis** | | ≥1 life-limiting condition: non-curable cancer; end-stage organ failure (cardiac, renal, liver, respiratory); neurodegenerative disease (including dementia); cerebrovascular disease; AIDS^2^[3]. | |  |
|  | **Disease status** | | Advanced disease: e.g. metastases, refractory ascites, ventilator dependence. Limited prognosis: life expectancy expected to be ≤12 months Deteriorating trajectory: functional decline, increasing symptom burden. Unstable events: frequent falls, recurrent hospitalisations, nursing home admission. | |  |
|  | **Treatment response** | | No active treatment offered, or poor response to prior disease-modifying therapy. | |  |
|  | **Healthcare use** | | High service utilisation: frequent hospital/outpatient visits, community crisis response. Palliative care involvement for symptom management or end-of-life planning. | |  |
|  | **Care focus** | | Documented shift to palliative care (symptom management, psychosocial support); evidence of advance care planning. | |  |
|  | **Symptom burden** | | Refractory symptoms not responsive to disease-modifying treatment (e.g. severe pain, dyspnoea, fatigue). | |  |
|  | **Nutrition/ hydration** | | Dysphagia or significant unintentional weight loss (>10% in 6 months); need for thickened fluids. | |  |
|  | **Caring needs** | | Frailty/functional dependence: frailty score >4, AKPS^3^ <70, housebound, or nursing home resident. Dependency for ADL^4^: formal care packages multiple times/day. Identified caregiver burden. | |  |
|  | **Benefit eligibility** | | Eligibility under state provisions: e.g. Special Rules (prognosis <1 month); Continuing Healthcare funding. | |  |
|  | **Indices/tools** | | Prognostic and needs-based tools: Charlson Comorbidity Index; Supportive and Palliative Care Indicators Tool (SPICT); NECPAL^5^; GSF^6^ eligibility; Disease Burden Morbidity Assessment^7^ (DBMA ≥10). | |  |

*^1^Multiple long-term conditions; ^2^Acquired Immune Deficiency Syndrome; ^3^Australia-modified Karnofsky performance status*; *^4^Activities of daily living*; *^5^Necesidades Paliativas (Palliative Needs tool);* *^6^Gold Standards Framework,^7^Disease Burden Morbidity Assessment*

1.	Barnett K, Mercer SW, Norbury M, Watt G, Wyke S, Guthrie B. Epidemiology of multimorbidity and implications for health care, research, and medical education: a cross-sectional study. *Lancet*. Jul 7 2012;380(9836):37–43. doi:10.1016/s0140-6736(12)60240-2

2.	Head A, Fleming K, Kypridemos C, Schofield P, Pearson-Stuttard J, O'Flaherty M. Inequalities in incident and prevalent multimorbidity in England, 2004&#x2013;19: a population-based, descriptive study. *The Lancet Healthy Longevity*. 2021;2(8):e489–e497. doi:10.1016/S2666-7568(21)00146-X

3.	Murtagh FEM, Bausewein C, Verne J, Groeneveld EI, Kaloki YE, Higginson IJ. How many people need palliative care? A study developing and comparing methods for population-based estimates. *Palliative Medicine*. 2013;28(1):49–58. doi:10.1177/0269216313489367
