# Supplementary material for: The palliative care needs of people with multiple long-term conditions and their family caregivers: a scoping review of mixed study designs
Source: Age Ageing. 2026 Nov 13;55(11):afag324. doi: 10.1093/ageing/afag324 (PMC13761345; doi:10.1093/ageing/afag324)
Supplement: Supplementary file 1 [file supplementary_material_afag324.zip › aa-26-1473-File006.docx]

The Palliative Care Needs of People with Multiple Long-Term Conditions (MLTC) and their Family Caregivers: A Scoping Review of Mixed Study Designs

## **Appendix 4: Database search strategies**

Database searches were conducted on 8 September 2025. The number of records retrieved from each database was:

- MEDLINE: 4014
- EMBASE: 4595
- CINAHL: 2926
- PsycINFO: 1043
- Scopus: 2367

Total records before deduplication: 14,945

After deduplication and cleaning: 7568

Detailed MEDLINE search strategy is provided below, with equivalent adaptations for other databases.

##### **Ovid MEDLINE**

Ovid MEDLINE(R) ALL <1946 to September 8, 2025>

| 1 | palliative care/ or terminal care/ or hospice care/ or "Hospice and Palliative Care Nursing"/ or Hospices/ |  |
| --- | --- | --- |
| 2 | Terminally Ill/ and (hospice* or palliat*).mp. |  |
| 3 | (hospice* or palliat* or "end of life").ti,kf. |  |
| 4 | hospice*.ab. /freq=2 or palliat*.ab. /freq=2 or "end of life".ab. /freq=2 |  |
| 5 | exp "aged, 80 and over"/ or frail elderly/ or Health Services for the Aged/ |  |
| 6 | or/1-5 |  |
| 7 | multiple chronic conditions/ |  |
| 8 | multimorbidity/ |  |
| 9 | *comorbidity/ |  |
| 10 | *Chronic Disease/ or (chronic* adj5 (conditions or ill*)).ti. or chronic disease.ti. |  |
| 11 | (comorbidity/ or comorbid*.ti,kf.) and (chronic disease/ or (chronic* adj5 (conditions or ill*)).kf. or chronic disease.kf.) |  |
| 12 | (multimorbid* or multi*-morbid* or multipathol* or multi-pathol* or polymorbid* or poly-morbid* or polypathol* or poly-pathol*).mp. |  |
| 13 | (multiple adj3 (chronic or advanced or complex or long-term or longterm or comorbid* or co-morbid* or ill* or disease* or condition* or syndrome* or disorder? or health problem*)).ti. |  |
| 14 | (multiple chronic or multiple long-term or multiple longterm).ab,kf. |  |
| 15 | (comorbidities or co-morbidities or comorbid).ti. |  |
| 16 | (multiple comorbid* or multiple co-morbid*).ti,ab,kf. |  |
| 17 | (complex chronic or chronic complex).ti,kf,ab. | |
| 18 | (complex adj3 (condition* or health problem*)).ti. | |
| 19 | (complex adj3 (needs or patient*)).ti. | |
| 20 | (complex adj3 (needs or patient* or conditions or health problem*)).mp. and complex*.ab. /freq=3 | |
| 21 | complexity.ti. or complexity.ab. /freq=2 | |
| 22 | (advanced adj3 conditions).ti. | |
| 23 | ((noncancer or non-cancer or nonmalignant or non-malignant or non-oncologic* or nononcologic*).ti. and (chronic* or longterm* or long-term* or comorbid* or complex* or burden*).mp.) or ((noncancer or non-cancer or nonmalignant or non-malignant or non-oncologic* or nononcologic*) adj2 (conditions or chronic* or longterm* or long-term* or comorbid*)).mp. | |
| 24 | ((Charlson or Elixhauer) and (comorbid* or index*)).ti,kf,ab. and (comorbidity/ or chronic disease/) |  |
| 25 | ((long-term or longterm) and (condition* or illness* or care or therap*)).ti. and ((palliative or "end of life").ti. or *palliative care/ or *terminal care/ or *hospice care/) |  |
| 26 | or/7-25 |  |
| 27 | (care need or needs or experiences).ti,kf. |  |
| 28 | ((experience or experiences) adj3 (care or healthcare or patient*)).ab. and (experience or experiences).ab. /freq=2 |  |
| 29 | changing needs.mp. |  |
| 30 | (complex adj3 needs).mp. |  |
| 31 | (patient* adj3 perspective*).ab. /freq=2 or (patient* adj3 needs).ab. /freq=2 |  |
| 32 | (symptom* adj3 burden*).mp. |  |
| 33 | ((illness* or conditions or symptom*) adj4 manag*).mp. |  |
| 34 | priorit*.ti. or priorit*.ab. /freq=3 |  |
| 35 | ((patient* or carer* or caregiver* or stakeholder*) adj5 perspective*).ti,kf. |  |
| 36 | (quality adj3 (healthcare or care)).ti,kf. or (quality adj3 care).ab. /freq=2 or (quality adj3 healthcare).ab. /freq=2 |  |
| 37 | (care adj2 need*).ab. /freq=2 |  |
| 38 | ((preference* or priorit* or strategies or experience or experiences or challenges) adj4 care).mp. and care.ab. /freq=2 |  |
| 39 | ((care or service*) adj2 ("use" or utili*)).ti. |  |
| 40 | ((needs or care or health*) adj10 trajector*).mp. or trajector*.ti. |  |
| 41 | ((patient or person) adj2 (centred or centered)).mp. |  |
| 42 | ((treatment or care or patient) adj3 pathway*).mp. |  |
| 43 | ("do not" adj3 "resuscit*").mp. |  |
| 44 | care plan*.ti,kf. or care plan*.ab. /freq=2 |  |
| 45 | (patient* adj2 manag*).mp. and manag*.ab. /freq=2 |  |
| 46 | (Karnofsky Performance or Palliative Performance Scale or Integrated Palliative Outcome Scale or Edmonton Symptom Assessment System or Barthel Index).af. |  |
| 47 | Karnofsky Performance Status/ |  |
| 48 | Chronic Disease/th or Multiple Chronic Conditions/th |  |
| 49 | Health Services Needs and Demand/ |  |
| 50 | needs assessment/ |  |
| 51 | symptom burden/ |  |
| 52 | Caregiver Burden/ |  |
| 53 | disease management/ |  |
| 54 | Patient Preference/ |  |
| 55 | Decision Making/ |  |
| 56 | clinical decision-making/ |  |
| 57 | clinical reasoning/ |  |
| 58 | patient care planning/ |  |
| 59 | Case Management/ |  |
| 60 | advance care planning/ |  |
| 61 | advance directives/ |  |
| 62 | living wills/ |  |
| 63 | Quality of Health Care/ |  |
| 64 | Program Evaluation/ |  |
| 65 | health services research/ |  |
| 66 | health care surveys/ |  |
| 67 | health impact assessment/ |  |
| 68 | patient reported outcome measures/ |  |
| 69 | Primary Health Care/ |  |
| 70 | Holistic Health/ |  |
| 71 | Life tables/ |  |
| 72 | Life Expectancy/ |  |
| 73 | quality-adjusted life years/ |  |
| 74 | Survival Rate/ |  |
| 75 | Referral and Consultation/ |  |
| 76 | Workload/ |  |
| 77 | exp "costs and cost analysis"/ |  |
| 78 | Caregivers/ and needs.mp. |  |
| 79 | Long-Term Care/ and care.ti. /freq=2 |  |
| 80 | functional status/ and care.mp. |  |
| 81 | *"Quality of Life"/ |  |
| 82 | *"Delivery of Health Care"/ |  |
| 83 | *Patient Care/ |  |
| 84 | or/27-83 |  |
| 85 | 6 and 26 and 84 |  |
| 86 | (multiple chronic or multiple longterm or multiple long term or multiple comorbid* or multiple co-morbid* or (multiple adj3 conditions) or ((chronic adj5 conditions) and complex*) or complex care needs or complex health needs).ti. |  |
| 87 | (multimorbid* or multi*-morbid* or polymorbid* or poly-morbid* or polypathol* or poly-pathol*).ti. and (multiple chronic conditions/ or multimorbidity/ or chronic disease/ or *comorbidity/ or care.ti. or multiple.ti.) |  |
| 88 | (complex adj3 needs).ti. and ((multimorbid* or multi*-morbid* or polymorbid* or poly-morbid* or polypathol* or poly-pathol* or multiple chronic or multiple long term or multiple longterm or (multiple adj4 conditions)).mp. or multiple chronic conditions/ or multimorbidity/) |  |
| 89 | (comorbid* or co-morbid* or complexity or (chronic adj5 conditions) or (complex adj5 (patient* or care*))).ti. and (multiple chronic conditions/ or multimorbidity/) |  |
| 90 | or/86-89 |  |
| 91 | *palliative care/ or *terminal care/ or *hospice care/ or *"Hospice and Palliative Care Nursing"/ or *Hospices/ or (hospice* or palliat* or "end of life").ti. |  |
| 92 | 85 or (90 and 84) or (90 and 91) |  |
| 93 | 1 or 2 or 7 or 8 or 13 or 18 or ((chronic disease/ or comorbidity/) and complex*.ti.) or (palliat* or hospice* or "end of life" or needs or trajector* or multimorbid* or multi*-morbid* or multipathol* or multi-pathol* or polymorbid* or poly-morbid* or polypathol* or poly-pathol*).ti. |  |
| 94 | 92 and 93 |  |
| 95 | limit 94 to ("all infant (birth to 23 months)" or "all child (0 to 18 years)" or "newborn infant (birth to 1 month)" or "infant (1 to 23 months)" or "preschool child (2 to 5 years)" or "child (6 to 12 years)" or "adolescent (13 to 18 years)") |  |
| 96 | limit 94 to ("all adult (19 plus years)" or "young adult (19 to 24 years)" or "adult (19 to 44 years)" or "young adult and adult (19-24 and 19-44)" or "middle age (45 to 64 years)" or "middle aged (45 plus years)" or "all aged (65 and over)" or "aged (80 and over)") |  |
| 97 | 94 not ((95 not 96) or child*.ti. or p?ediatr*.ti. or infant.ti. or infants.ti.) |  |
| 98 | limit 97 to (comment or editorial or news) |  |
| 99 | limit 97 to "review articles" and (review.ti. or "this* review".ab. or "this* systematic review".ab.) |  |
| 100 | 97 not (98 or 99) |  |

##### **Embase**

Embase <1974 to 2025 September 8>

| 1 | palliative therapy/ or terminal care/ or hospice care/ or palliative nursing/ or hospice nursing/ or hospice patient/ or hospice/ |
| --- | --- |
| 2 | terminally ill patient/ and (hospice* or palliat*).mp. |
| 3 | (hospice* or palliat* or "end of life").ti,kf. |
| 4 | hospice*.ab. /freq=2 or palliat*.ab. /freq=2 or "end of life".ab. /freq=2 |
| 5 | very elderly/ or frail elderly/ or elderly care/ |
| 6 | or/1-5 |
| 7 | multiple chronic conditions/ |
| 8 | *comorbidity/ |
| 9 | *chronic disease/ or (chronic* adj5 (conditions or ill*)).ti. or chronic disease.ti. |
| 10 | (comorbidity/ or comorbid*.ti,kf.) and (chronic disease/ or (chronic* adj5 (conditions or ill*)).kf. or chronic disease.kf.) |
| 11 | (multimorbid* or multi*-morbid* or multipathol* or multi-pathol* or polymorbid* or poly-morbid* or polypathol* or poly-pathol*).mp. |
| 12 | (multiple adj3 (chronic or advanced or complex or long-term or longterm or comorbid* or co-morbid* or ill* or disease* or condition* or syndrome* or disorder? or health problem*)).ti. |
| 13 | (multiple chronic or multiple long-term or multiple longterm).ab,kf. |
| 14 | (comorbidities or co-morbidities or comorbid).ti. |
| 15 | (multiple comorbid* or multiple co-morbid*).ti,ab,kf. |
| 16 | (complex chronic or chronic complex).ti,kf,ab. |
| 17 | (complex adj3 (condition* or health problem*)).ti. |
| 18 | (complex adj3 (needs or patient*)).ti. |
| 19 | (complex adj3 (needs or patient* or conditions or health problem*)).mp. and complex*.ab. /freq=3 |
| 20 | complexity.ti. or complexity.ab. /freq=2 |
| 21 | (advanced adj3 conditions).ti. |
| 22 | ((noncancer or non-cancer or nonmalignant or non-malignant or non-oncologic* or nononcologic*).ti. and (chronic* or longterm* or long-term* or comorbid* or complex* or burden*).mp.) or ((noncancer or non-cancer or nonmalignant or non-malignant or non-oncologic* or nononcologic*) adj2 (conditions or chronic* or longterm* or long-term* or comorbid*)).mp. |
| 23 | (((Charlson or Elixhauer) and (comorbid* or index*)).ti,kf,ab. and (comorbidity/ or chronic disease/)) or *Charlson Comorbidity Index/ |
| 24 | ((long-term or longterm) and (condition* or illness* or care or therap*)).ti. and ((palliative or "end of life").ti. or *palliative therapy/ or *terminal care/ or *hospice care/) |
| 25 | or/7-24 |
| 26 | (care need or needs or experiences).ti,kf. |
| 27 | ((experience or experiences) adj3 (care or healthcare or patient*)).ab. and (experience or experiences).ab. /freq=2 |
| 28 | changing needs.mp. |
| 29 | (complex adj3 needs).mp. |
| 30 | (patient* adj3 perspective*).ab. /freq=2 or (patient* adj3 needs).ab. /freq=2 |
| 31 | (symptom* adj3 burden*).mp. |
| 32 | ((illness* or conditions or symptom*) adj4 manag*).mp. |
| 33 | priorit*.ti. or priorit*.ab. /freq=3 |
| 34 | ((patient* or carer* or caregiver* or stakeholder*) adj5 perspective*).ti,kf. |
| 35 | (quality adj3 (healthcare or care)).ti,kf. or (quality adj3 care).ab. /freq=2 or (quality adj3 healthcare).ab. /freq=2 |
| 36 | (care adj2 need*).ab. /freq=2 |
| 37 | ((preference* or priorit* or strategies or experience or experiences or challenges) adj4 care).mp. and care.ab. /freq=2 |
| 38 | ((care or service*) adj2 ("use" or utili*)).ti. |
| 39 | ((needs or care or health*) adj10 trajector*).mp. or trajector*.ti. |
| 40 | ((patient or person) adj2 (centred or centered)).mp. |
| 41 | ((treatment or care or patient) adj3 pathway*).mp. |
| 42 | ("do not" adj3 "resuscit*").mp. |
| 43 | care plan*.ti,kf. or care plan*.ab. /freq=2 |
| 44 | (patient* adj2 manag*).mp. and manag*.ab. /freq=2 |
| 45 | (Karnofsky Performance or Palliative Performance Scale or Integrated Palliative Outcome Scale or Edmonton Symptom Assessment System or Barthel Index).af. |
| 46 | functional status assessment/ or karnofsky performance status/ |
| 47 | multiple chronic conditions/dm, th or chronic disease/dm, th |
| 48 | health care need/ or medical care need/ or supportive care need/ or unmet medical need/ |
| 49 | needs assessment/ |
| 50 | caregiver burden/ |
| 51 | caregiver support/ |
| 52 | patient preference/ |
| 53 | decision making/ |
| 54 | medical decision making/ |
| 55 | clinical decision making/ |
| 56 | disease management/ |
| 57 | case management/ |
| 58 | "escalation of care"/ |
| 59 | treatment planning/ |
| 60 | patient care planning/ |
| 61 | health care planning/ |
| 62 | advance care planning/ |
| 63 | living will/ |
| 64 | nursing care plan/ |
| 65 | health care quality/ |
| 66 | health care surveillance/ |
| 67 | health care survey/ |
| 68 | health impact assessment/ |
| 69 | patient-reported outcome/ |
| 70 | "quality of nursing care"/ |
| 71 | primary health care/ |
| 72 | holistic care/ |
| 73 | life table/ |
| 74 | life expectancy/ |
| 75 | quality adjusted life year/ |
| 76 | survival rate/ |
| 77 | patient referral/ |
| 78 | workload/ |
| 79 | exp economic evaluation/ |
| 80 | exp program evaluation/ |
| 81 | caregiver/ and needs.mp. |
| 82 | functional status/ and care.mp. |
| 83 | long term care/ and care.ti. /freq=2 |
| 84 | *"quality of life"/ |
| 85 | *health care delivery/ |
| 86 | *health care utilization/ |
| 87 | *patient care/ |
| 88 | or/26-87 |
| 89 | 6 and 25 and 88 |
| 90 | (multiple chronic or multiple longterm or multiple long term or multiple comorbid* or multiple co-morbid* or (multiple adj3 conditions) or ((chronic adj5 conditions) and complex*) or complex care needs or complex health needs).ti. |
| 91 | (multimorbid* or multi*-morbid* or polymorbid* or poly-morbid* or polypathol* or poly-pathol*).ti. and (multiple chronic conditions/ or chronic disease/ or *comorbidity/ or care.ti. or multiple.ti.) |
| 92 | (complex adj3 needs).ti. and ((multimorbid* or multi*-morbid* or polymorbid* or poly-morbid* or polypathol* or poly-pathol* or multiple chronic or multiple long term or multiple longterm or (multiple adj4 conditions)).mp. or multiple chronic conditions/) |
| 93 | (comorbid* or co-morbid* or complexity or (chronic adj5 conditions) or (complex adj5 (patient* or care*))).ti. and multiple chronic conditions/ |
| 94 | or/90-93 |
| 95 | *palliative therapy/ or *terminal care/ or *palliative nursing/ or *hospice care/ or *hospice/ or *hospice nursing/ or *hospice patient/ or (hospice* or palliat* or "end of life").ti. |
| 96 | 89 or (94 and 88) or (94 and 95) |
| 97 | 1 or 2 or 7 or 12 or 17 or palliat*.ti. or hospice*.ti. or ((chronic disease/ or comorbidity/) and complex*.ti.) or (palliat* or hospice* or "end of life" or needs or trajector* or multimorbid* or multi*-morbid* or multipathol* or multi-pathol* or polymorbid* or poly-morbid* or polypathol* or poly-pathol*).ti. |
| 98 | 96 and 97 |
| 99 | limit 98 to (embryo or infant or child or preschool child <1 to 6 years> or school child <7 to 12 years> or adolescent <13 to 17 years>) |
| 100 | limit 98 to (adult <18 to 64 years> or aged <65+ years>) |
| 101 | 98 not ((99 not 100) or child*.ti. or p?ediatr*.ti. or infant.ti. or infants.ti.) |
| 102 | limit 101 to (conference abstract or editorial or note) |
| 103 | limit 101 to "review" and (review.ti. or "this* review".ab. or "this* systematic review".ab.) |
| 104 | 101 not (102 or 103) |

##### **CINAHL**

Search modes – Proximity; Interface - EBSCOhost Research Databases; Search Screen - Advanced Search; Database - CINAHL

| S1 | MH ("Palliative Care" OR "Palliative Care Nursing" OR "Palliative Care Nurses" OR "Palliative Medicine" OR "Hospice and Palliative Nurses Association" OR "Hospice Nurses" OR "Terminal Care" OR "Hospice Care" OR "Hospice Patients") |
| --- | --- |
| S2 | MH ("Terminally Ill Patients") AND (hospice* OR palliat*) |
| S3 | TI (hospice* OR palliat* OR "end of life") |
| S4 | AB ((hospice* AND palliat*) OR (palliat* AND "end of life") OR (hospice* AND "end of life")) |
| S5 | MH ("Aged, 80 and Over+" OR "Frail Elderly" OR "Health Services for Older Persons") |
| S6 | S1 OR S2 OR S3 OR S4 OR S5 |
| S7 | MM "Comorbidity" |
| S8 | MM "Chronic Disease" OR TI ((chronic* N5 (conditions OR ill*)) OR TI "chronic disease" |
| S9 | (MH "Comorbidity" OR TI comorbid*) AND MH "Chronic Disease" |
| S10 | (multimorbid* OR multi*-morbid* OR multipathol* OR multi-pathol* OR polymorbid* OR poly-morbid* OR polypathol* OR poly-pathol*) |
| S11 | TI (multiple N3 (chronic OR advanced OR complex OR "long-term" OR longterm OR comorbid* OR co-morbid* OR ill* OR disease* OR condition* OR syndrome* OR disorder* OR "health problem*")) |
| S12 | TI (comorbidities OR "co-morbidities" OR comorbid OR (advanced N3 conditions) OR complexity) |
| S13 | ("multiple comorbid*" OR "multiple co-morbid*" OR "complex chronic" OR "chronic complex" OR "multiple chronic" OR "multiple long-term" OR "multiple longterm") |
| S14 | TI (complex N3 (needs OR patient* OR condition* OR "health problem*")) |
| S15 | (complex N3 (needs OR patient* OR conditions OR "health problem*")) AND TI complex* |
| S16 | (TI (noncancer OR non-cancer OR nonmalignant OR non-malignant OR non-oncologic* OR nononcologic*) AND (chronic* OR longterm* OR long-term* OR comorbid* OR complex* OR burden*)) OR ((noncancer OR non-cancer OR nonmalignant OR non-malignant OR non-oncologic* OR nononcologic*) N2 (conditions OR chronic* OR longterm* OR long-term* OR comorbid*)) |
| S17 | ((Charlson OR Elixhauer) AND (comorbid* OR index*)) AND (MH "comorbidity" OR MH "chronic disease") |
| S18 | TI ((long-term OR longterm) AND (condition* OR illness* OR care OR therap*)) AND (TI (palliative OR "end of life") OR MM "palliative care" OR MM "terminal care" OR MM "hospice care" OR MM "palliative medicine") |
| S19 | S7 OR S8 OR S9 OR S10 OR S11 OR S12 OR S13 OR S14 OR S15 OR S16 OR S17 OR S18 |
| S20 | TI ("care need" OR needs OR experiences OR priorit* OR trajector* OR "care plan*") |
| S21 | ("changing needs" OR (complex N3 needs) OR (symptom* N3 burden*) OR ("do not" N3 "resuscit*")OR "care need*") |
| S22 | TI ((patient* OR carer* OR caregiver* OR stakeholder*) N5 perspective*) |
| S23 | TI (quality N3 (healthcare OR care)) |
| S24 | TI ((care OR service*) N2 ("use" OR utili*)) |
| S25 | ((illness* OR conditions OR symptom*) N4 manag*) |
| S26 | ((preference* OR priorit* OR strategies OR experience OR experiences OR challenges) N4 care) |
| S27 | ((needs OR care OR health*) N10 trajector*) |
| S28 | ((patient OR person) N2 (centred OR centered)) |
| S29 | ((treatment OR care OR patient) N3 pathway*) |
| S30 | ("Karnofsky Performance" OR "Palliative Performance Scale" OR "Integrated Palliative Outcome Scale" OR "Edmonton Symptom Assessment System" OR "Barthel Index") |
| S31 | MH ("Karnofsky Performance Status" OR "Chronic Disease/TH" OR "Health Services Needs and Demand" OR "Needs Assessment" OR "Symptom Burden" OR "Caregiver Burden" OR "Disease Management" OR "Patient Preference" OR "Decision Making" OR MH "Decision Making, Patient" OR "Decision Making, Clinical" OR "Clinical Reasoning" OR "Patient Care Plans" OR MH "Nursing Care Plans" OR MH "Nursing Care Plans, Computerized" OR "Case Management" OR MH "Case Management Nursing" OR "Advance Care Planning" OR "Advance Directives" OR "Living Wills " OR MH "Resuscitation Orders" OR "Quality of Health Care" OR "Program Evaluation" OR "Health Services Research" OR MH " Quality of Care Research" OR "Health Impact Assessment" OR "Patient-Reported Outcomes" OR "Primary Health Care" OR "Holistic Health" OR "Life Table Method" OR "Life Expectancy" OR "Quality-Adjusted Life Years" OR "Survival Rate" OR "Referral and Consultation" OR "Workload" OR "Costs and Cost Analysis+") |
| S32 | MH "Caregivers" AND needs |
| S33 | MH ("Long Term Care" OR " Long Term Care Nursing") AND TI care |
| S34 | MH "Functional Status" AND care |
| S35 | MM ("Quality of Life" OR "Health Care Delivery" OR "Patient Care") |
| S36 | S20 OR S21 OR S22 OR S23 OR S24 OR S25 OR S26 OR S27 OR S28 OR S29 OR S30 OR S31 OR S32 OR S33 OR S34 OR S35 |
| S37 | S6 AND S19 AND S36 |
| S38 | TI ("multiple chronic" OR "multiple longterm" OR "multiple long term" OR "multiple comorbid*" OR "multiple co-morbid*" OR (multiple N3 conditions) OR ((chronic N5 conditions) AND complex*) OR "complex care needs" OR "complex health needs") |
| S39 | TI (multimorbid* OR multi*-morbid* OR polymorbid* OR poly-morbid* OR polypathol* OR poly-pathol*) AND (MH "chronic disease" OR MM "comorbidity" OR TI (care OR multiple)) |
| S40 | TI (complex N3 needs) AND ((multimorbid* OR "multi*-morbid*" OR polymorbid* OR poly-morbid* OR polypathol* OR poly-pathol* OR "multiple chronic" OR "multiple long term" OR "multiple longterm" OR (multiple N4 conditions))) |
| S41 | TI (comorbid* OR co-morbid* OR complexity OR (chronic N5 conditions) OR (complex N5 (patient* OR care*))) AND MH comorbidity AND MH "chronic disease" |
| S42 | S38 OR S39 OR S40 OR S41 |
| S43 | MM ("Palliative Care" OR "Palliative Care Nursing" OR "Palliative Medicine" OR "Terminal Care" OR "Hospice Care" OR "Hospice Patients") OR TI (hospice* OR palliat* OR "end of life") |
| S44 | S37 OR (S42 AND S36) OR (S42 AND S43) |
| S45 | S1 OR S2 OR S11 OR TI (complex N3 (condition* OR "health problem*")) OR (MH ("chronic disease" OR comorbidity) AND TI complex*) OR TI (needs OR trajector* OR palliat* OR hospice* OR "end of life" OR multimorbid* OR multi*-morbid* OR multipathol* OR multi-pathol* OR polymorbid* OR poly-morbid* OR polypathol* OR poly-pathol*) |
| S46 | (S1 OR S2 OR S11 OR (MH ("chronic disease" OR comorbidity) AND TI complex*) OR TI (needs OR trajector* OR palliat* OR hospice* OR multimorbid* OR multi*-morbid* OR multipathol* OR multi-pathol* OR polymorbid* OR poly-morbid* OR polypathol* OR poly-pathol*)) AND (S44 AND S45) |
| S47 | S46 NOT ((MH ("Adolescence+" OR "Child+" OR "Minors (Legal)") NOT MH "Adult+") OR TI (paediatr* OR pediatr* OR infant OR infants OR child*)) |
| S48 | S47 [Limiters - Publication Type: Commentary, Editorial] |
| S49 | S47 AND (TI review OR AB ("this* review" OR "this* systematic review") [Limiters - Publication Type: Meta Analysis, Meta Synthesis, Review, Systematic Review] |
| S50 | S47 NOT (S48 OR S49) |

##### **APA PsycInfo**

APA PsycInfo <1806 to September Week 2 2025>

| 1 | palliative care/ or hospice/ |
| --- | --- |
| 2 | terminally ill patients/ and (hospice* or palliat*).mp. |
| 3 | (hospice* or palliat* or "end of life").ti,id. |
| 4 | hospice*.ab. /freq=2 or palliat*.ab. /freq=2 or "end of life".ab. /freq=2 |
| 5 | elder care/ or geriatric patients/ |
| 6 | or/1-5 |
| 7 | comorbidity/ |
| 8 | chronic illness/ or (chronic* adj5 (conditions or ill*)).ti. or chronic disease.ti. |
| 9 | (comorbidity/ or comorbid*.ti,id.) and (chronic illness/ or (chronic* adj5 (conditions or ill*)).id. or chronic disease.id.) |
| 10 | (multimorbid* or multi*-morbid* or multipathol* or multi-pathol* or polymorbid* or poly-morbid* or polypathol* or poly-pathol*).mp. |
| 11 | (multiple adj3 (chronic or advanced or complex or long-term or longterm or comorbid* or co-morbid* or ill* or disease* or condition* or syndrome* or disorder? or health problem*)).ti. |
| 12 | (multiple chronic or multiple long-term or multiple longterm).ab,id. |
| 13 | (comorbidities or co-morbidities or comorbid).ti. |
| 14 | (multiple comorbid* or multiple co-morbid*).ti,ab,id. |
| 15 | (complex chronic or chronic complex).ti,id,ab. |
| 16 | (complex adj3 (condition* or health problem*)).ti. |
| 17 | (complex adj3 (needs or patient*)).ti. |
| 18 | (complex adj3 (needs or patient* or conditions or health problem*)).mp. and complex*.ab. /freq=3 |
| 19 | complexity.ti. or complexity.ab. /freq=2 |
| 20 | (advanced adj3 conditions).ti. |
| 21 | ((noncancer or non-cancer or nonmalignant or non-malignant or non-oncologic* or nononcologic*).ti. and (chronic* or longterm* or long-term* or comorbid* or complex* or burden*).mp.) or ((noncancer or non-cancer or nonmalignant or non-malignant or non-oncologic* or nononcologic*) adj2 (conditions or chronic* or longterm* or long-term* or comorbid*)).mp. |
| 22 | ((Charlson or Elixhauer) and (comorbid* or index*)).ti,id,ab. and (comorbidity/ or chronic illness/) |
| 23 | ((long-term or longterm) and (condition* or illness* or care or therap*)).ti. and ((palliative or "end of life").ti. or *palliative care/ or *terminal care/ or *hospice care/) |
| 24 | or/7-23 |
| 25 | (care need or needs or experiences).ti,id. |
| 26 | ((experience or experiences) adj3 (care or healthcare or patient*)).ab. and (experience or experiences).ab. /freq=2 |
| 27 | changing needs.mp. |
| 28 | (complex adj3 needs).mp. |
| 29 | (patient* adj3 perspective*).ab. /freq=2 or (patient* adj3 needs).ab. /freq=2 |
| 30 | (symptom* adj3 burden*).mp. |
| 31 | ((illness* or conditions or symptom*) adj4 manag*).mp. |
| 32 | priorit*.ti. or priorit*.ab. /freq=3 |
| 33 | ((patient* or carer* or caregiver* or stakeholder*) adj5 perspective*).ti,id. |
| 34 | (quality adj3 (healthcare or care)).ti,id. or (quality adj3 care).ab. /freq=2 or (quality adj3 healthcare).ab. /freq=2 |
| 35 | (care adj2 need*).ab. /freq=2 |
| 36 | ((preference* or priorit* or strategies or experience or experiences or challenges) adj4 care).mp. and care.ab. /freq=2 |
| 37 | ((care or service*) adj2 ("use" or utili*)).ti. |
| 38 | ((needs or care or health*) adj10 trajector*).mp. or trajector*.ti. |
| 39 | ((patient or person) adj2 (centred or centered)).mp. |
| 40 | ((treatment or care or patient) adj3 pathway*).mp. |
| 41 | ("do not" adj3 "resuscit*").mp. |
| 42 | care plan*.ti,id. or care plan*.ab. /freq=2 |
| 43 | (patient* adj2 manag*).mp. and manag*.ab. /freq=2 |
| 44 | (Karnofsky Performance or Palliative Performance Scale or Integrated Palliative Outcome Scale or Edmonton Symptom Assessment System or Barthel Index).af. |
| 45 | health service needs/ |
| 46 | needs assessment/ |
| 47 | caregiver burden/ |
| 48 | managed care/ |
| 49 | health maintenance organizations/ |
| 50 | disease management/ |
| 51 | preferences/ |
| 52 | decision making/ |
| 53 | treatment planning/ or discharge planning/ or posttreatment followup/ |
| 54 | client treatment matching/ |
| 55 | advance directives/ |
| 56 | "quality of care"/ |
| 57 | program evaluation/ |
| 58 | patient reported outcome measures/ |
| 59 | primary health care/ |
| 60 | holistic health/ |
| 61 | life expectancy/ |
| 62 | professional referral/ or client transfer/ |
| 63 | work load/ |
| 64 | "quality of life"/ or "health related quality of life"/ |
| 65 | health care services/ |
| 66 | health care delivery/ |
| 67 | health care utilization/ |
| 68 | caregiving/ |
| 69 | functional status/ |
| 70 | long term care/ and care.ti. /freq=2 |
| 71 | caregivers/ and needs.ti. |
| 72 | exp "costs and cost analysis"/ |
| 73 | or/25-72 |
| 74 | 6 and 24 and 73 |
| 75 | (multiple chronic or multiple longterm or multiple long term or multiple comorbid* or multiple co-morbid* or (multiple adj3 conditions) or ((chronic adj5 conditions) and complex*) or complex care needs or complex health needs).ti. |
| 76 | (multimorbid* or multi*-morbid* or polymorbid* or poly-morbid* or polypathol* or poly-pathol*).ti. and (chronic illness/ or comorbidity/ or care.ti. or multiple.ti.) |
| 77 | (complex adj3 needs).ti. and (multimorbid* or multi*-morbid* or polymorbid* or poly-morbid* or polypathol* or poly-pathol* or multiple chronic or multiple long term or multiple longterm or (multiple adj4 conditions)).mp. |
| 78 | or/75-77 |
| 79 | palliative care/ or hospice/ or (hospice* or palliat* or "end of life").ti. |
| 80 | 74 or (78 and 73) or (78 and 79) |
| 81 | 1 or 2 or 11 or 16 or ((chronic illness/ or comorbidity/) and complex*.ti.) or (palliat* or hospice* or "end of life" or needs or trajector* or multimorbid* or multi*-morbid* or multipathol* or multi-pathol* or polymorbid* or poly-morbid* or polypathol* or poly-pathol*).ti. |
| 82 | 80 and 81 |
| 83 | limit 82 to (100 childhood <birth to age 12 yrs> or 120 neonatal <birth to age 1 mo> or 140 infancy <2 to 23 mo> or 160 preschool age <age 2 to 5 yrs> or 180 school age <age 6 to 12 yrs> or 200 adolescence <age 13 to 17 yrs>) |
| 84 | limit 82 to ("300 adulthood <age 18 yrs and older>" or 320 young adulthood <age 18 to 29 yrs> or 340 thirties <age 30 to 39 yrs> or 360 middle age <age 40 to 64 yrs> or "380 aged <age 65 yrs and older>" or "390 very old <age 85 yrs and older>") |
| 85 | 82 not ((83 not 84) or child*.ti. or p?ediatr*.ti. or infant.ti. or infants.ti.) |
| 86 | limit 85 to (chapter or editorial or "column/opinion" or "comment/reply" or dissertation) |
| 87 | limit 85 to reviews and (review.ti. or "this* review".ab. or "this* systematic review".ab.) |
| 88 | 85 not (86 or 87) |

##### **Scopus**

(( ( TITLE ( multimorbid* OR multi*-morbid* OR multipathol* OR multi-pathol* OR polymorbid* OR poly-morbid* OR polypathol* OR poly-pathol* OR ( multiple W/3 ( chronic OR advanced OR complex OR long-term OR longterm OR comorbid* OR co-morbid* OR ill* OR disease* OR condition* OR syndrome* OR disorder* OR "health problem*" ) ) OR needs OR trajector* OR palliat* OR hospice* OR "end of life" ) ) AND ( ( ( TITLE ( hospice* OR palliat* OR "end of life" ) ) AND ( ( TITLE ( "multiple chronic" OR "multiple longterm" OR "multiple long term" OR "multiple comorbid*" OR "multiple co-morbid*" OR ( multiple W/3 conditions ) OR ( ( chronic W/5 conditions ) AND complex* ) OR "complex care needs" OR "complex health needs" ) ) OR ( TITLE ( ( multimorbid* OR multi*-morbid* OR polymorbid* OR poly-morbid* OR polypathol* OR poly-pathol* ) AND ( "chronic disease" OR comorbidity OR care OR multiple ) ) ) OR ( TITLE ( complex W/3 needs ) AND TITLE-ABS-KEY ( ( multimorbid* OR "multi*-morbid*" OR polymorbid* OR poly-morbid* OR polypathol* OR poly-pathol* OR "multiple chronic" OR "multiple long term" OR "multiple longterm" OR ( multiple W/4 conditions ) ) ) ) ) ) OR ( ( ( TITLE ( "multiple chronic" OR "multiple longterm" OR "multiple long term" OR "multiple comorbid*" OR "multiple co-morbid*" OR ( multiple W/3 conditions ) OR ( ( chronic W/5 conditions ) AND complex* ) OR "complex care needs" OR "complex health needs" ) ) OR ( TITLE ( ( multimorbid* OR multi*-morbid* OR polymorbid* OR poly-morbid* OR polypathol* OR poly-pathol* ) AND ( "chronic disease" OR comorbidity OR care OR multiple ) ) ) OR ( TITLE ( complex W/3 needs ) AND TITLE-ABS-KEY ( ( multimorbid* OR "multi*-morbid*" OR polymorbid* OR poly-morbid* OR polypathol* OR poly-pathol* OR "multiple chronic" OR "multiple long term" OR "multiple longterm" OR ( multiple W/4 conditions ) ) ) ) ) AND ( ( TITLE-ABS-KEY ( "care need*" OR "changing needs" OR ( complex W/3 needs ) OR ( symptom* W/3 burden* ) OR ( ( illness* OR conditions OR symptom* ) W/4 manag* ) OR ( ( preference* OR priorit* OR strategies OR experience OR experiences OR challenges ) W/4 care ) OR ( ( needs OR care OR health* ) W/10 trajector* ) OR ( ( patient OR person ) W/2 ( centred OR centered ) ) OR ( ( treatment OR care OR patient ) W/3 pathway* ) OR ( "do not" W/3 "resuscit*" ) OR "Karnofsky Performance" OR "Palliative Performance Scale" OR "Integrated Palliative Outcome Scale" OR "Edmonton Symptom Assessment System" OR "Barthel Index" ) ) OR ( TITLE ( needs OR experiences OR ( patient* W/3 perspective* ) OR priorit* OR ( ( patient* OR carer* OR caregiver* OR stakeholder* ) W/5 perspective* ) OR ( quality W/3 ( healthcare OR care ) ) OR ( ( care OR service* ) W/2 ( "use" OR utili* ) ) OR "care plan*" OR trajector* ) ) ) ) OR ( ( ( TITLE-ABS-KEY ( "care need*" OR "changing needs" OR ( complex W/3 needs ) OR ( symptom* W/3 burden* ) OR ( ( illness* OR conditions OR symptom* ) W/4 manag* ) OR ( ( preference* OR priorit* OR strategies OR experience OR experiences OR challenges ) W/4 care ) OR ( ( needs OR care OR health* ) W/10 trajector* ) OR ( ( patient OR person ) W/2 ( centred OR centered ) ) OR ( ( treatment OR care OR patient ) W/3 pathway* ) OR ( "do not" W/3 "resuscit*" ) OR "Karnofsky Performance" OR "Palliative Performance Scale" OR "Integrated Palliative Outcome Scale" OR "Edmonton Symptom Assessment System" OR "Barthel Index" ) ) OR ( TITLE ( needs OR experiences OR ( patient* W/3 perspective* ) OR priorit* OR ( ( patient* OR carer* OR caregiver* OR stakeholder* ) W/5 perspective* ) OR ( quality W/3 ( healthcare OR care ) ) OR ( ( care OR service* ) W/2 ( "use" OR utili* ) ) OR "care plan*" OR trajector* ) ) ) AND ( ( TITLE ( "chronic disease" OR ( chronic* W/5 ( conditions OR ill* ) ) OR ( multiple W/3 ( chronic OR advanced OR complex OR "long-term" OR longterm OR comorbid* OR co-morbid* OR ill* OR disease* OR condition* OR syndrome* OR disorder* OR "health problem*" ) ) OR comorbidities OR "co-morbidities" OR comorbid OR ( complex W/3 ( condition* OR "health problem*" OR needs OR patient* ) ) OR complexity OR ( advanced W/3 conditions ) ) ) OR ( TITLE-ABS-KEY ( multimorbid* OR multi*-morbid* OR multipathol* OR multi-pathol* OR polymorbid* OR poly-morbid* OR polypathol* OR poly-pathol* OR "multiple chronic" OR "multiple long-term" OR "multiple longterm" OR "multiple comorbid*" OR "multiple co-morbid*" OR "complex chronic" OR "chronic complex" ) ) OR ( ( TITLE ( comorbid* ) OR KEY ( comorbid* ) ) AND KEY ( ( chronic* W/5 ( conditions OR ill* ) ) OR "chronic disease" ) ) OR ( TITLE-ABS-KEY ( complex W/3 ( needs OR patient* OR conditions OR "health problem*" ) ) AND TITLE ( complex* ) ) OR ( ( TITLE ( noncancer OR non-cancer OR nonmalignant OR non-malignant OR non-oncologic* OR nononcologic* ) AND TITLE-ABS-KEY ( chronic* OR longterm* OR long-term* OR comorbid* OR complex* OR burden* ) ) OR TITLE-ABS-KEY ( ( noncancer OR non-cancer OR nonmalignant OR non-malignant OR non-oncologic* OR nononcologic* ) W/2 ( conditions OR chronic* OR longterm* OR long-term* OR comorbid* ) ) ) OR ( TITLE-ABS-KEY ( ( charlson OR elixhauer ) AND ( comorbid* OR index* ) ) AND ( KEY ( comorbid* OR "chronic disease" ) OR TITLE ( comorbid* ) ) ) OR ( TITLE ( ( long-term OR longterm ) AND ( condition* OR illness* OR care OR therap* ) ) AND ( TITLE ( palliat* OR "end of life" ) OR KEY ( palliat* OR "end of life" ) ) ) ) AND ( TITLE ( hospice* OR palliat* OR "end of life" ) OR KEY ( hospice* OR palliat* OR "end of life" ) OR ABS ( ( hospice* AND palliat* ) OR ( palliat* AND "end of life" ) OR ( hospice* AND "end of life" ) ) OR TITLE ( older OR elder* OR ( frail* AND aged ) ) ) ) ) ) AND NOT ( TITLE ( paediatr* OR pediatr* OR infant OR infants OR child* ) OR ( DOCTYPE ( re ) AND ( TITLE ( review ) OR ABS ( "this* review" OR "this* systematic review" ) ) ) OR DOCTYPE ( cp ) OR DOCTYPE ( no ) OR DOCTYPE ( ed ) OR DOCTYPE ( ch ) OR DOCTYPE ( bk ) ))
