# Supplementary material for: The palliative care needs of people with multiple long-term conditions and their family caregivers: a scoping review of mixed study designs
Source: Age Ageing. 2026 Nov 13;55(11):afag324. doi: 10.1093/ageing/afag324 (PMC13761345; doi:10.1093/ageing/afag324)
Supplement: Supplementary file 1 [file supplementary_material_afag324.zip › aa-26-1473-File008.docx]

The Palliative Care Needs of People with Multiple Long-Term Conditions (MLTC) and their Family Caregivers: A Scoping Review of Mixed Study Designs

## **Appendix 6: Studies with domain and subdomain mapping**

|  | **COMPREHENSIVE SUPPORT NEEDS  IN THE CONTEXT OF FUNCTIONAL DECLINE** | | | | | | | **PROACTIVE CARE NEEDS SHAPED BY LIMITED DEATH LITERACY AND PROGNOSTIC UNCERTAINTY** | | | **SYSTEMS-LEVEL NEEDS FOR COORDINATION AND COLLABORATION** | | | **COMPOUNDED NEEDS ARISING FROM INEQUALITY** | |
| --- | --- | --- | --- | --- | --- | --- | --- | --- | --- | --- | --- | --- | --- | --- | --- |
|  | **Functional needs** | **Physical needs** | **Psychological needs** | **Spiritual needs** | **Social needs** | **Financial needs** | **Family/ Carer needs** | **Advance Care Planning needs** | **Goal Concordant Care needs** | **Prognostic Uncertainty / Episodic Deterioration / Reactive Care** | **Communication needs** | **Coordination needs** | **MDT/ Collaboration needs** | **Diagnostic inequality** | **Socio-demographic inequality** |
| Abedini et al., 2020 | ✅ | ✅ | ✅ | ❌ | ✅ | ❌ | ✅ | ✅ | ❌ | ✅ | ❌ | ❌ | ❌ | ❌ | ✅ |
| Almagro et al., 2020 | ✅ | ✅ | ✅ | ❌ | ✅ | ❌ | ✅ | ✅ | ✅ | ✅ | ❌ | ✅ | ✅ | ❌ | ✅ |
| Alnajar et al., 2025 | ✅ | ✅ | ✅ | ✅ | ✅ | ✅ | ❌ | ❌ | ❌ | ✅ | ✅ | ✅ | ✅ | ✅ | ✅ |
| Barrio-Cortes et al., 2021 | ✅ | ✅ | ✅ | ❌ | ✅ | ❌ | ✅ | ❌ | ❌ | ✅ | ❌ | ✅ | ✅ | ❌ | ✅ |
| Beach et al., 2020 | ✅ | ✅ | ✅ | ❌ | ✅ | ❌ | ✅ | ❌ | ❌ | ✅ | ❌ | ✅ | ✅ | ❌ | ✅ |
| Bekker et al., 2022 | ❌ | ❌ | ✅ | ✅ | ✅ | ❌ | ❌ | ✅ | ✅ | ✅ | ✅ | ✅ | ❌ | ✅ | ❌ |
| Bernabeu-Wittel et al., 2010 | ✅ | ✅ | ✅ | ❌ | ✅ | ❌ | ❌ | ❌ | ❌ | ✅ | ❌ | ✅ | ✅ | ❌ | ✅ |
| Boyd et al., 2019 | ✅ | ✅ | ✅ | ❌ | ✅ | ❌ | ✅ | ✅ | ✅ | ✅ | ✅ | ✅ | ❌ | ✅ | ✅ |
| Bushor and Rowser, 2015 | ❌ | ✅ | ❌ | ❌ | ❌ | ✅ | ❌ | ✅ | ❌ | ✅ | ❌ | ✅ | ❌ | ✅ | ✅ |
| Calsina-Berna et al., 2022 | ✅ | ✅ | ✅ | ❌ | ✅ | ❌ | ❌ | ✅ | ❌ | ✅ | ❌ | ✅ | ❌ | ✅ | ✅ |
| Campos-Calderón et al., 2016 | ❌ | ✅ | ❌ | ❌ | ❌ | ❌ | ❌ | ✅ | ❌ | ✅ | ❌ | ✅ | ❌ | ✅ | ❌ |
| Castany et al., 2025 | ❌ | ✅ | ❌ | ❌ | ❌ | ❌ | ❌ | ❌ | ❌ | ❌ | ❌ | ❌ | ❌ | ❌ | ❌ |
| Cenzer et al., 2025 | ✅ | ✅ | ✅ | ❌ | ❌ | ❌ | ❌ | ❌ | ❌ | ❌ | ❌ | ❌ | ❌ | ❌ | ✅ |
| Chan and Pang, 2006 | ✅ | ✅ | ✅ | ✅ | ✅ | ❌ | ❌ | ✅ | ❌ | ✅ | ✅ | ❌ | ❌ | ✅ | ✅ |
| Chen et al., 2024 | ❌ | ❌ | ❌ | ❌ | ❌ | ❌ | ❌ | ✅ | ❌ | ✅ | ✅ | ✅ | ❌ | ✅ | ✅ |
| Chen et al., 2025 | ✅ | ❌ | ❌ | ❌ | ❌ | ❌ | ❌ | ❌ | ❌ | ✅ | ❌ | ❌ | ❌ | ❌ | ❌ |
| Chochinov et al., 2016 | ✅ | ✅ | ✅ | ✅ | ✅ | ❌ | ❌ | ❌ | ❌ | ✅ | ❌ | ❌ | ❌ | ✅ | ✅ |
| Clarke et al., 2012 | ✅ | ✅ | ✅ | ✅ | ✅ | ❌ | ✅ | ✅ | ✅ | ✅ | ✅ | ✅ | ❌ | ❌ | ✅ |
| Cummings et al., 2018 | ✅ | ✅ | ✅ | ❌ | ❌ | ❌ | ✅ | ✅ | ❌ | ✅ | ✅ | ✅ | ✅ | ✅ | ✅ |
| Cunningham et al., 2022 | ✅ | ✅ | ✅ | ❌ | ✅ | ✅ | ✅ | ✅ | ✅ | ✅ | ✅ | ✅ | ✅ | ✅ | ✅ |
| Dahlqvist et al., 2019 | ❌ | ❌ | ❌ | ❌ | ❌ | ❌ | ✅ | ✅ | ✅ | ✅ | ✅ | ✅ | ✅ | ✅ | ✅ |
| Disler et al., 2024 | ❌ | ✅ | ❌ | ✅ | ❌ | ❌ | ✅ | ✅ | ✅ | ✅ | ✅ | ✅ | ✅ | ✅ | ✅ |
| Ebenau et al., 2019 | ✅ | ✅ | ✅ | ✅ | ✅ | ✅ | ✅ | ✅ | ✅ | ✅ | ✅ | ✅ | ✅ | ✅ | ✅ |
| Esteban-Burgos et al., 2021 | ✅ | ✅ | ✅ | ❌ | ✅ | ❌ | ❌ | ❌ | ❌ | ✅ | ❌ | ❌ | ❌ | ✅ | ❌ |
| Etkind et al., 2025 | ✅ | ❌ | ✅ | ❌ | ❌ | ❌ | ✅ | ✅ | ✅ | ✅ | ✅ | ✅ | ✅ | ❌ | ❌ |
| Finucane et al., 2017 | ❌ | ❌ | ❌ | ❌ | ❌ | ❌ | ❌ | ❌ | ❌ | ❌ | ✅ | ❌ | ✅ | ✅ | ❌ |
| Finucane et al., 2021 | ✅ | ❌ | ❌ | ❌ | ❌ | ❌ | ❌ | ✅ | ❌ | ✅ | ❌ | ❌ | ❌ | ❌ | ✅ |
| Francis et al., 2020 | ✅ | ✅ | ✅ | ✅ | ✅ | ✅ | ✅ | ❌ | ✅ | ✅ | ✅ | ✅ | ✅ | ❌ | ✅ |
| Frazer and Mobley, 2017 | ✅ | ✅ | ✅ | ❌ | ✅ | ❌ | ✅ | ❌ | ✅ | ✅ | ✅ | ❌ | ✅ | ❌ | ❌ |
| Gill et al., 2014 | ❌ | ✅ | ✅ | ❌ | ❌ | ❌ | ✅ | ❌ | ✅ | ✅ | ✅ | ✅ | ✅ | ❌ | ❌ |
| Ginggeaw et al., 2025 | ✅ | ✅ | ✅ | ❌ | ✅ | ❌ | ❌ | ❌ | ❌ | ❌ | ❌ | ❌ | ❌ | ❌ | ❌ |
| Gothe et al., 2020 | ❌ | ✅ | ❌ | ❌ | ❌ | ❌ | ❌ | ❌ | ❌ | ❌ | ✅ | ❌ | ❌ | ✅ | ✅ |
| Greenle et al., 2019 | ❌ | ✅ | ❌ | ❌ | ❌ | ❌ | ❌ | ✅ | ✅ | ❌ | ✅ | ❌ | ✅ | ✅ | ❌ |
| Haines et al., 2019 | ❌ | ❌ | ❌ | ❌ | ❌ | ❌ | ✅ | ✅ | ✅ | ❌ | ✅ | ❌ | ❌ | ❌ | ✅ |
| Harrison et al., 2023 | ✅ | ✅ | ✅ | ✅ | ❌ | ❌ | ✅ | ❌ | ✅ | ✅ | ✅ | ✅ | ✅ | ✅ | ✅ |
| Hash et al., 2016 | ❌ | ❌ | ✅ | ❌ | ❌ | ❌ | ❌ | ✅ | ❌ | ❌ | ❌ | ❌ | ❌ | ❌ | ✅ |
| Holley et al., 2009 | ✅ | ✅ | ✅ | ❌ | ✅ | ❌ | ✅ | ✅ | ✅ | ✅ | ✅ | ✅ | ✅ | ✅ | ✅ |
| Hung et al., 2013 | ✅ | ✅ | ✅ | ❌ | ❌ | ❌ | ✅ | ✅ | ❌ | ✅ | ✅ | ✅ | ✅ | ❌ | ✅ |
| Kang et al., 2014 | ❌ | ❌ | ❌ | ❌ | ❌ | ❌ | ✅ | ❌ | ❌ | ✅ | ❌ | ✅ | ✅ | ✅ | ❌ |
| Kasdorf et al., 2022 | ❌ | ❌ | ❌ | ❌ | ❌ | ❌ | ✅ | ✅ | ❌ | ✅ | ✅ | ✅ | ✅ | ✅ | ✅ |
| Kato et al., 2025 | ✅ | ❌ | ❌ | ❌ | ❌ | ❌ | ❌ | ❌ | ❌ | ❌ | ❌ | ❌ | ❌ | ❌ | ✅ |
| Kramer and Auer, 2005 | ✅ | ✅ | ✅ | ✅ | ✅ | ❌ | ✅ | ✅ | ✅ | ❌ | ✅ | ✅ | ✅ | ❌ | ✅ |
| Kramer, 2013 | ✅ | ❌ | ✅ | ❌ | ✅ | ✅ | ✅ | ✅ | ✅ | ✅ | ✅ | ✅ | ✅ | ✅ | ✅ |
| Kricke et al., 2019 | ❌ | ✅ | ✅ | ✅ | ❌ | ❌ | ✅ | ❌ | ❌ | ❌ | ✅ | ✅ | ❌ | ❌ | ✅ |
| Lee et al., 2016 | ✅ | ✅ | ✅ | ❌ | ✅ | ✅ | ✅ | ❌ | ❌ | ✅ | ✅ | ❌ | ❌ | ❌ | ✅ |
| Llop-Medina et al., 2022 | ✅ | ✅ | ✅ | ✅ | ✅ | ✅ | ✅ | ✅ | ❌ | ✅ | ✅ | ✅ | ✅ | ✅ | ✅ |
| Luk et al., 2013 | ✅ | ✅ | ❌ | ❌ | ❌ | ✅ | ❌ | ✅ | ❌ | ✅ | ❌ | ✅ | ❌ | ✅ | ✅ |
| Luo et al., 2025 | ❌ | ✅ | ✅ | ❌ | ❌ | ❌ | ❌ | ❌ | ❌ | ✅ | ❌ | ❌ | ❌ | ❌ | ✅ |
| Maloney et al., 2025 | ❌ | ❌ | ✅ | ❌ | ✅ | ❌ | ✅ | ❌ | ❌ | ❌ | ❌ | ❌ | ❌ | ✅ | ✅ |
| Marcucci et al., 2016 | ✅ | ✅ | ✅ | ❌ | ✅ | ✅ | ✅ | ❌ | ❌ | ✅ | ✅ | ✅ | ✅ | ✅ | ✅ |
| Marventano et al., 2016 | ❌ | ✅ | ✅ | ❌ | ✅ | ❌ | ✅ | ✅ | ✅ | ✅ | ✅ | ✅ | ✅ | ✅ | ✅ |
| Mason et al., 2013 | ✅ | ✅ | ✅ | ❌ | ✅ | ❌ | ✅ | ✅ | ❌ | ✅ | ✅ | ✅ | ❌ | ✅ | ❌ |
| Mason et al., 2016 | ✅ | ✅ | ✅ | ❌ | ✅ | ❌ | ❌ | ❌ | ❌ | ❌ | ❌ | ❌ | ❌ | ❌ | ✅ |
| McDermott et al., 2021 | ❌ | ❌ | ❌ | ❌ | ❌ | ❌ | ❌ | ✅ | ✅ | ✅ | ✅ | ✅ | ❌ | ❌ | ❌ |
| McGhan et al., 2013 | ✅ | ❌ | ❌ | ❌ | ❌ | ❌ | ✅ | ❌ | ❌ | ❌ | ✅ | ✅ | ✅ | ❌ | ❌ |
| McKittrick et al., 2025 | ✅ | ✅ | ✅ | ❌ | ✅ | ✅ | ✅ | ❌ | ❌ | ✅ | ✅ | ✅ | ✅ | ❌ | ✅ |
| McParland et al., 2025 | ✅ | ✅ | ✅ | ❌ | ✅ | ✅ | ✅ | ❌ | ❌ | ✅ | ✅ | ✅ | ❌ | ❌ | ✅ |
| Méndez-Bailón et al., 2022 | ✅ | ✅ | ✅ | ❌ | ❌ | ❌ | ❌ | ❌ | ❌ | ❌ | ❌ | ❌ | ❌ | ❌ | ❌ |
| Mota-Romero et al., 2021 | ✅ | ✅ | ✅ | ❌ | ✅ | ❌ | ✅ | ❌ | ❌ | ✅ | ❌ | ✅ | ✅ | ✅ | ✅ |
| Murali et al., 2021 | ✅ | ✅ | ✅ | ❌ | ❌ | ❌ | ❌ | ❌ | ❌ | ❌ | ❌ | ❌ | ❌ | ❌ | ❌ |
| Murali et al., 2023a | ✅ | ✅ | ❌ | ❌ | ❌ | ❌ | ❌ | ❌ | ❌ | ✅ | ❌ | ❌ | ❌ | ✅ | ✅ |
| Murali et al., 2023b | ✅ | ✅ | ✅ | ❌ | ❌ | ❌ | ❌ | ❌ | ❌ | ❌ | ❌ | ❌ | ❌ | ❌ | ✅ |
| Ng et al., 2020 | ✅ | ❌ | ❌ | ❌ | ❌ | ❌ | ❌ | ❌ | ❌ | ❌ | ❌ | ✅ | ❌ | ❌ | ❌ |
| Nicholson et al., 2018 | ✅ | ✅ | ✅ | ✅ | ✅ | ❌ | ✅ | ✅ | ❌ | ✅ | ✅ | ✅ | ✅ | ✅ | ✅ |
| Nkhoma et al., 2025 | ✅ | ✅ | ✅ | ✅ | ✅ | ✅ | ✅ | ❌ | ✅ | ❌ | ✅ | ✅ | ✅ | ❌ | ✅ |
| Nothelle et al., 2022 | ✅ | ✅ | ❌ | ❌ | ❌ | ❌ | ❌ | ❌ | ❌ | ✅ | ❌ | ✅ | ❌ | ❌ | ❌ |
| Parker et al., 2005 | ✅ | ✅ | ✅ | ✅ | ✅ | ❌ | ❌ | ❌ | ❌ | ✅ | ✅ | ✅ | ✅ | ✅ | ✅ |
| Piers et al., 2010 | ❌ | ❌ | ❌ | ❌ | ❌ | ❌ | ❌ | ✅ | ✅ | ✅ | ✅ | ✅ | ✅ | ❌ | ✅ |
| Pop et al., 2023 | ✅ | ✅ | ✅ | ❌ | ✅ | ✅ | ✅ | ❌ | ❌ | ✅ | ✅ | ✅ | ✅ | ✅ | ✅ |
| Portz et al., 2017 | ✅ | ✅ | ✅ | ❌ | ❌ | ❌ | ❌ | ❌ | ❌ | ❌ | ❌ | ✅ | ✅ | ❌ | ❌ |
| Reinke et al., 2019 | ✅ | ✅ | ✅ | ❌ | ✅ | ❌ | ❌ | ❌ | ❌ | ✅ | ✅ | ✅ | ✅ | ✅ | ✅ |
| Reis Júnior et al., 2024 | ✅ | ✅ | ✅ | ❌ | ❌ | ❌ | ❌ | ❌ | ❌ | ❌ | ❌ | ❌ | ❌ | ❌ | ❌ |
| Robinson et al., 2022 | ✅ | ✅ | ✅ | ❌ | ✅ | ❌ | ✅ | ❌ | ❌ | ✅ | ✅ | ✅ | ✅ | ❌ | ✅ |
| Rodríguez-Álvaro et al., 2022 | ✅ | ✅ | ✅ | ❌ | ✅ | ❌ | ✅ | ❌ | ❌ | ✅ | ✅ | ✅ | ✅ | ✅ | ✅ |
| Romanò et al., 2022 | ✅ | ✅ | ✅ | ❌ | ✅ | ❌ | ✅ | ❌ | ❌ | ✅ | ✅ | ✅ | ✅ | ✅ | ✅ |
| Rosella et al., 2014 | ✅ | ✅ | ✅ | ❌ | ✅ | ✅ | ❌ | ❌ | ❌ | ✅ | ✅ | ✅ | ✅ | ✅ | ✅ |
| Safabakhsh et al., 2023 | ✅ | ✅ | ✅ | ✅ | ✅ | ✅ | ✅ | ❌ | ❌ | ✅ | ✅ | ✅ | ✅ | ✅ | ✅ |
| Salinas et al., 2023 | ✅ | ✅ | ✅ | ✅ | ✅ | ✅ | ✅ | ❌ | ❌ | ✅ | ✅ | ✅ | ✅ | ✅ | ✅ |
| Santaeugènia et al., 2021 | ✅ | ✅ | ✅ | ❌ | ✅ | ❌ | ✅ | ✅ | ✅ | ✅ | ✅ | ✅ | ✅ | ✅ | ✅ |
| Schellinger et al., 2017 | ✅ | ✅ | ✅ | ✅ | ✅ | ✅ | ✅ | ✅ | ✅ | ✅ | ✅ | ✅ | ✅ | ❌ | ✅ |
| Skilbeck et al., 2017 | ✅ | ✅ | ✅ | ✅ | ✅ | ❌ | ❌ | ❌ | ✅ | ✅ | ✅ | ✅ | ✅ | ❌ | ✅ |
| Sri-on et al., 2024 | ✅ | ✅ | ✅ | ✅ | ✅ | ✅ | ✅ | ✅ | ✅ | ✅ | ✅ | ✅ | ✅ | ✅ | ✅ |
| Suriyawongpaisal et al., 2019 | ✅ | ✅ | ✅ | ❌ | ✅ | ❌ | ✅ | ❌ | ✅ | ✅ | ✅ | ✅ | ✅ | ❌ | ✅ |
| Tagliafico et al., 2023 | ✅ | ✅ | ✅ | ❌ | ✅ | ❌ | ✅ | ❌ | ✅ | ✅ | ✅ | ✅ | ✅ | ✅ | ✅ |
| Tan et al., 2013 | ✅ | ✅ | ✅ | ✅ | ✅ | ❌ | ✅ | ✅ | ✅ | ✅ | ✅ | ✅ | ✅ | ✅ | ✅ |
| Tanuseputro et al., 2016 | ✅ | ✅ | ❌ | ❌ | ✅ | ❌ | ✅ | ❌ | ❌ | ✅ | ✅ | ✅ | ✅ | ✅ | ✅ |
| Tschirhart et al., 2014 | ✅ | ✅ | ❌ | ❌ | ❌ | ❌ | ❌ | ✅ | ✅ | ✅ | ✅ | ✅ | ✅ | ✅ | ✅ |
| Wagner et al., 2019 | ✅ | ✅ | ❌ | ❌ | ❌ | ❌ | ❌ | ✅ | ✅ | ✅ | ✅ | ✅ | ✅ | ❌ | ✅ |
| Wu et al., 2020 | ✅ | ✅ | ✅ | ✅ | ✅ | ✅ | ✅ | ❌ | ❌ | ✅ | ✅ | ✅ | ✅ | ❌ | ✅ |
| Wu-Jun et al., 2014 | ✅ | ✅ | ✅ | ✅ | ✅ | ✅ | ✅ | ✅ | ✅ | ✅ | ✅ | ✅ | ✅ | ❌ | ✅ |
