# Supplementary material for: The palliative care needs of people with multiple long-term conditions and their family caregivers: a scoping review of mixed study designs
Source: Age Ageing. 2026 Nov 13;55(11):afag324. doi: 10.1093/ageing/afag324 (PMC13761345; doi:10.1093/ageing/afag324)
Supplement: Supplementary file 1 [file supplementary_material_afag324.zip › aa-26-1473-File009.docx]

The Palliative Care Needs of People with Multiple Long-Term Conditions (MLTC) and their Family Caregivers: A Scoping Review of Mixed Study Designs

**Appendix 7. Example of the analytical synthesis process**

The review used a hierarchical process in which study findings were first coded at the extract level, then interpreted as a need (sub-domain), before being synthesised into broader conceptual domains. The examples below illustrate how individual findings from Beach et al. (2020) were synthesised into the domain *Comprehensive Support Needs in the Context of Functional Decline*.

| **Study finding (data extract)** | **Descriptive code** | **Interpretive code** | **Need  (sub-domain)** | **Main domain** |
| --- | --- | --- | --- | --- |
| *Despite caregivers’ effort to support the needs of their care-recipients, care-recipients still report adverse consequences of unmet needs (p.468).* | Existing caregiving arrangements insufficient to meet care needs. | Older people experience unmet care needs despite family involvement, suggesting requirement for additional caregiver resources, support, and capacity. | Need for family/ carer support | **Comprehensive support needs in the context of functional decline** |
| *Over a third of those meeting all three criteria reported wet or soiled clothing in the past month due to unmet toileting needs (p. 464).* | Inadequate assistance with toileting and activities of daily living. | Functional dependency not being adequately supported, resulting in adverse consequences. | Need for functional support | **Comprehensive support needs in the context of functional decline** |

Findings were coded according to their underlying meaning rather than solely their descriptive content. In the first example, the extract highlighted persistent unmet needs despite the presence of family caregivers. The emphasis was therefore interpreted as a requirement for additional support for caregivers and coded as a *Need for family/carer support*. In the second example, the consequence of unmet assistance with toileting was interpreted as evidence of inadequate support with activities of daily living and coded as a *Need for functional support*. Both codes reflected wider support needs associated with frailty, disability, and increasing functional dependence, and so these sub-domains were synthesised within the overarching domain *Comprehensive Support Needs in the Context of Functional Decline*.
