# Supplementary material for: The palliative care needs of people with multiple long-term conditions and their family caregivers: a scoping review of mixed study designs
Source: Age Ageing. 2026 Nov 13;55(11):afag324. doi: 10.1093/ageing/afag324 (PMC13761345; doi:10.1093/ageing/afag324)
Supplement: Supplementary file 1 [file supplementary_material_afag324.zip › aa-26-1473-File010.docx]

The Palliative Care Needs of People with Multiple Long-Term Conditions (MLTC) and their Family Caregivers: A Scoping Review of Mixed Study Designs

**Appendix 8. Detailed methods**

## Design and reporting

A scoping review was selected because the review question concerned the extent, range and nature of a large and heterogeneous body of evidence, and the aim was to identify gaps and provide a foundation for future practice, policy‑relevant research, and more focused evidence synthesis [22]. The review adhered to PRISMA-ScR guidelines [23,24] (Appendix 1). The protocol was registered with PROSPERO (registration number: CRD42024510220), with deviations from the protocol reported in Appendix 2. Deviations were: a change of design from a systematic review to a scoping review, after preliminary searching showed marked heterogeneity in populations, MLTC definitions, settings and outcomes; the subsequent omission of formal critical appraisal; narrowing of the population to adults 50 years and over; addition of a requirement for at least one palliative phase indicator; restriction to original empirical studies and removal of the planned grey literature component; extension of the search end date; and the addition of a second stage of synthesis. Rationales for each are given in Appendix 2. Quantitative, qualitative and mixed-methods primary studies were eligible. Critical appraisal was not undertaken, consistent with scoping review guidance; the review therefore describes what has been reported and does not appraise the strength or reliability of that reporting [25].

## Eligibility criteria

Inclusion criteria were defined by characterising the “palliative phase” as a shift towards holistic symptom management and quality-of-life optimisation towards end-of-life [6]. Definitions of advanced MLTC were synthesised from the literature [5,10,11,26-29]. Studies were eligible if they reported original empirical research (quantitative, qualitative or mixed methods) on the needs of adults aged 50 years and over with two or more long-term conditions and at least one indicator of a palliative phase, or on the needs of their adult caregivers, in any setting or country. Indicators of a palliative phase comprised a life-limiting diagnosis, advanced disease, a prognosis of 12 months or less, a deteriorating trajectory, high service use or dependency, a documented shift to palliative care, or identification by a prognostic or needs-based tool such as SPICT or NECPAL. Studies were excluded if they addressed a single index condition, early-stage MLTC without indicators of advanced disease, or reported no need-related data; and where MLTC populations could not be disaggregated, or the mean or median age of the MLTC population was 49 years or below. The age threshold was set below the conventional 65 years so as not to exclude populations in whom MLTC develops earlier: onset occurs 10 to 15 years earlier in the most deprived compared with the most affluent areas [30], and the median age of onset has fallen while socioeconomic inequalities have widened, particularly below 65 years [31]. Study eligibility criteria are detailed in Appendix 3.

## Search strategy

A comprehensive search of MEDLINE, EMBASE, CINAHL, Scopus, and PsycINFO was conducted from inception to 8 September 2025. Only peer-reviewed publications were included; conference abstracts and preprints were followed up with authors. Search strategies were developed with an information specialist. The strategy combined two core concepts, MLTC (including multimorbidity, complex chronic conditions and comorbidity index terms) and palliative or end-of-life care, with a third concept covering needs and care experience, using subject headings and free-text terms. Search strings for each database, with the date run and records retrieved, are provided in Appendix 4.

## Study selection

Records were imported and de-duplicated in Endnote [32] by an information specialist (removed) and then imported into Rayyan [33], where all records underwent dual independent screening at both title/abstract and full-text stages. Full texts were retrieved via institutional subscriptions and library document supply, with interlibrary loan and direct requests to authors where necessary; five records could not be obtained (main manuscript, Figure 1). Screening was conducted in Rayyan by the reviewers (FD, OF, AI, EEJ, SB, LR, KE-S, CS, TPK). Calibration occurred iteratively during title and abstract screening, with reviewers discussing conflicts, clarifying eligibility criteria, and ensuring consistency in study selection. This process continued until a shared understanding of the inclusion and exclusion criteria was established. Discrepancies were resolved through discussion and adjudication by a third reviewer (BH), a senior member of the team with expertise in palliative and end-of-life care research [23].

## Data charting

Data were extracted and charted by OF and MA, and verified by FD.  A standardised data extraction form developed specifically for this review was used. The form was piloted on a sample of included studies by two reviewers, with modifications made to improve clarity and completeness before full data extraction commenced. Further iterative changes were made during the process of preparing this manuscript, for example to include groupings of data, or where additional data was charted. Charting captured author, year, country and World Bank income classification; study design and care setting, both as reported and as grouped; primary study aim; number of eligible MLTC participants; sex distribution; mean or median age and age range of the MLTC cohort; whether race or ethnicity data were collected, and whether analysed beyond descriptive or covariate use; the comorbidity measure or MLTC definition used; whether an ICD version was used for coding; and the presence or absence of each of the 15 needs subdomains. Where data could not be disaggregated from a wider study sample, this is recorded as not available in Table 1 and Appendix 5.

## Data synthesis

Findings from each included study, whether reported as themes, survey responses, instrument scores or routinely collected data, were coded inductively to descriptive statements of need, compared across studies and grouped into 15 subdomains; these were refined iteratively and then applied to all 90 studies to produce the coverage matrix in Appendix 6. All 90 studies contributed to the synthesis and to the coverage matrix, and none was excluded.

Quantitative and qualitative findings were integrated using a convergent integrated approach, in which quantitative results were narratively described and combined with qualitative findings against a common set of subdomains [34,35]. The 15 subdomains were then grouped into four overarching domains, using published conceptual frameworks in geriatrics and palliative care as an organising lens, with a worked example of the analytical synthesis process in Appendix 7.

Illustrative studies were selected to represent diverse designs (qualitative, quantitative, mixed methods), settings (community, hospital, long-term care, hospice), and geographies (high-income and LMIC settings). Selection aimed to capture major illness trajectories (cancer, organ failure, dementia/frailty, and broader MLTC) and information-rich data (large cohorts, multi-site audits, or in-depth qualitative accounts). This was not intended to imply comparative study quality, but to illustrate typical or distinctive manifestations of each subdomain.
